# Looking for the mechanism of arsenate respiration of *Fusibacter* sp. strain 3D3, independent of ArrAB

**DOI:** 10.3389/fmicb.2022.1029886

**Published:** 2022-12-01

**Authors:** Mauricio Acosta-Grinok, Susana Vázquez, Nicolás Guiliani, Sabrina Marín, Cecilia Demergasso

**Affiliations:** ^1^Biotechnology Center, Universidad Católica del Norte, Antofagasta, Chile; ^2^Cátedra de Biotecnología, Facultad de Farmacia y Bioquímica, Universidad de Buenos Aires, Buenos Aires, Argentina; ^3^Instituto de Nanobiotecnología (NANOBIOTEC), Universidad de Buenos Aires (UBA) - Consejo Nacional de Investigaciones Científicas y Técnicas (CONICET), Buenos Aires, Argentina; ^4^Departamento de Biología, Facultad de Ciencias, Universidad de Chile, Antofagasta, Chile; ^5^Nucleus for the Study of Cancer at a Basic, Applied, and Clinical Level, Universidad Católica del Norte, Antofagasta, Chile

**Keywords:** *Fusibacter*, arsenic respiration, electron bifurcation, Rnf complex, ferredoxin, thioredoxin, *etf*, Northern Chile

## Abstract

The literature has reported the isolation of arsenate-dependent growing microorganisms which lack a canonical homolog for respiratory arsenate reductase, ArrAB. We recently isolated an arsenate-dependent growing bacterium from volcanic arsenic-bearing environments in Northern Chile, *Fusibacter* sp. strain 3D3 (*Fas*) and studied the arsenic metabolism in this Gram-positive isolate. Features of *Fas* deduced from genome analysis and comparative analysis with other arsenate-reducing microorganisms revealed the lack of ArrAB coding genes and the occurrence of two *arsC* genes encoding for putative cytoplasmic arsenate reductases named ArsC-1 and ArsC-2. Interestingly, ArsC-1 and ArsC-2 belong to the thioredoxin-coupled family (because of the redox-active disulfide protein used as reductant), but they conferred differential arsenate resistance to the *E. coli* WC3110 Δ*arsC* strain. PCR experiments confirmed the absence of *arrAB* genes and results obtained using uncouplers revealed that *Fas* growth is linked to the proton gradient. In addition, *Fas* harbors ferredoxin-NAD^+^ oxidoreductase (Rnf) and electron transfer flavoprotein (*etf*) coding genes. These are key molecular markers of a recently discovered flavin-based electron bifurcation mechanism involved in energy conservation, mainly in anaerobic metabolisms regulated by the cellular redox state and mostly associated with cytoplasmic enzyme complexes. At least three electron-bifurcating flavoenzyme complexes were evidenced in *Fas*, some of them shared in conserved genomic regions by other members of the *Fusibacter* genus. These physiological and genomic findings permit us to hypothesize the existence of an uncharacterized arsenate-dependent growth metabolism regulated by the cellular redox state in the *Fusibacter* genus.

## Introduction

The occurrence of an active As biogeochemical cycle in sediment samples of Salar de Ascotán, Northern Chile, has been previously confirmed by our group, by performing enrichment cultures (Lara et al., 2012). In addition, 16S rDNA sequencing analysis revealed that, despite being members of genera that had not been previously reported as As metabolizing, several isolates obtained from that salt flat are capable to metabolize As. Interestingly, one of these isolates belongs to the *Fusibacter* genus and we reported it as *Fusibacter* sp. strain 3D3 (hereafter called *Fas*; Serrano et al., 2017). In addition, by a preliminary analysis of the draft sequence of *Fas* genome we have revealed for the first time in a *Fusibacter* species the presence of an arsenate reductase gene (*arsC*) and of all the genes encoding the Rnf complex, and suggested the absence of genes encoding ArrAB proteins involved in dissimilatory arsenate reduction (Serrano et al., 2017).

ArsC is a cytoplasmic arsenate reductase reducing As(V) to As(III), which is then extruded out of the cell by the specific pumps. Three different ArsC prokaryotic families have been defined based on their protein structures, reduction mechanisms and location of the catalytic cysteine residues (Villadangos et al., 2011): (i) the glutathione (GSH)/glutaredoxin (Grx)-coupled class in Gram-negative bacteria such as *Escherichia coli* (Mukhopadhyay and Rosen, 2002); (ii) the thioredoxin (Trx)/thioredoxin reductase (TR)-dependent class in Gram-positive bacteria (Zegers et al., 2001); (iii) the mycothiol (MSH)/mycoredoxin (Mrx)-dependent class also in Gram-positive bacteria such as *Actinobacteria* spp. (Ordonez et al., 2009). Kinetic data on arsenate reduction have shown higher catalytic efficiency in Trx-linked arsenate reductases than in Grx-linked ones. In some cases, the efflux of As(III) can also be coupled to the electrochemical proton gradient, where chemical energy in the form of ATP is used to pump As(III) with the help of the ATPase ArsA (Rosen and Liu, 2009).

The number and type (Trx or Grx clade) of *arsC* genes present in the genomes of prokaryotic organisms is related to As resistance levels (Li and Krumholz, 2007; Achour-Rokbani et al., 2010; Cuebas et al., 2011; Villadangos et al., 2011). The Trx reducing system has been reported to be the most efficient system exploited by arsenate reductases (Villadangos et al., 2011). Besides, arsenate reductases with the same structural fold but depending on two different thiol-disulfide relay mechanisms (Trx and GSH) have also been observed in a single bacterial species, *Corynebacterium glutamicum* ATCC 13032 (Villadangos et al., 2011). In that case, a different role has been proposed for both enzymes, representatives of different families of prokaryotic ArsC: the Trx-dependent one would reduce arsenate to regulate the gene expression of the other, that is involved in the As resistance (Villadangos et al., 2011). A predominance of Trx-linked ArsC have been found in low G + C Gram-positive bacteria (Messens and Silver, 2006), which is the predominant bacterial group that we found in the volcanic As-impacted environments of Northern Chile (Lara et al., 2012; Escudero et al., 2013).

The absence of homologs of the respiratory arsenate reductase genes, a*rrAB*, has been reported for different isolated microorganisms. In *Desulfomicrobium* strain Ben-RB, neither ArrA nor ArsC were evidenced by PCR, and a cytochrome with increased activity in cultures with As(V) was proposed as the responsible for arsenate reduction (Macy et al., 2000). In *Pyrobaculum aerophilum,* a tetrathionate reductase (*ttrA*), highly expressed by culturing in presence of As(V), was hypothesized as a novel type of respiratory arsenate reductase (Cozen et al., 2009). In *Citrobacter* TSA, the authors reported that only *arsC* mRNA was strongly expressed when it was cultured with As(V), and hypothesized the occurrence of a linked electron flow to the cytoplasmic ArsC protein (Blum et al., 2018).

Interestingly, similar gaps in the energy metabolism of anaerobes (Bertsch et al., 2013; Buckel and Thauer, 2013) were closed by the characterization of Flavin-Based Electron Bifurcation (FBEB). This type of energy conservation is based on two main components: (i) the electron-bifurcating (electron transfer) flavoprotein complexes (Etf-complexes), where ferredoxins and flavodoxins act as low-potential terminal acceptors, and (ii) the electron transport phosphorylation (ETP) with protons (ferredoxin-proton reductase, Ech) or NAD^+^ (ferredoxin-NAD^+^ reductase, Rnf) as electron acceptors, where ferredoxins and flavodoxins re-oxidation drive electrochemical H^+^ and Na^+^ pumps (Schuchmann and Müller, 2016). Up to 12 Etf multienzyme complexes have been reported (Buckel and Thauer, 2013; Peters et al., 2018; Poudel et al., 2018; Liang et al., 2019). Almost all are located in the cytoplasm and are involved in the electron bifurcation (referred to as electron confurcation when operating in reverse) mechanism and associated to energy conservation. Furthermore, the studies on the distribution of the identified FBEB enzymes have shown that they are predominantly present among members of the *Firmicutes* and contribute to diverse metabolic pathways (Poudel et al., 2018; Liang et al., 2019). A key role in balancing the ratio of oxidized to reduced NAD(H) and ferredoxin (Fd) pools has been proposed for the FBEB mechanism. The presence of FBEB enzymes has also been identified in arsenate reducers (e.g., *Alkaliphilus oremlandii* OhILAs) but its activity was not clarified under As reduction conditions (Poudel et al., 2018).

Then, as we have previously reported that *Fas* possesses key molecular elements for As respiration (Serrano et al., 2017), the aim of this work was to assess the dependence of *Fas* growth on As(V) reduction and to elucidate the role of the cytoplasmic ArsC and the Rnf complex in this energy metabolism by culturing, revisiting its genome, and genetic approaches. In that way, we expect to contribute to the understanding of the means to achieve arsenate respiration in ArrAB-independent microorganisms, abundant in As bearing volcanic environments.

## Materials and methods

### Bacterial strains

*Fusibacter* sp. strain 3D3 was isolated at the Centro de Biotecnología, Universidad Católica del Norte, Antofagasta, Chile, from samples collected in the hypersaline sediments of the Salar de Ascotán in Northern Chile and deposited in the American Type Culture Collection as *Fusibacter ascotence* ATCC BAA-2418 (hereinafter referred to as *Fas*). The necessary tests and deposits to describe the isolate as a new species are running, and “*Fusibacter ascotence*” is the proposed name. The *Fas* draft genome assembly (Serrano et al., 2017) is available on NCBI (RefSeq GCF_001748365.1, GenBank GCA_001748365.1). The *E. coli* WC3110 Δ*arsC* strain was generously given by Dr. Barry P. Rosen.

### Culture characterization

All growth experiments were performed in duplicate in serum bottles containing 20 ml liquid Newman-modified minimal medium with lactate (10 mM), sulfate (20 mM), arsenate (2 mM), yeast extract (0.1%), NaCl (10 g L^−1^), and cysteine (1 mM), pH 7, inoculated with 1×10^6^ cells mL^−1^ from a fresh culture and incubated at 30°C in an anaerobic chamber under N_2_:CO_2_:H_2_ gas atmosphere (80:15:5 v/v) for 5 to 10 days in the dark, unless otherwise stated. An abiotic control was carried out in sterile medium without inoculum. Cell growth was monitored by microscope cell counting using a Neubauer improved chamber (0.01 mm x 0.0025 mm^2^, Marienfeld). To test for growth in the presence of oxygen, aerobic cultures were performed in shaking flasks incubated at 100 rpm in a rotatory shaker. The range of temperature for growth was tested between 15°C and 37°C, and the range of pH between 4 and 9. To assess the fermentative metabolism, *Fas* was grown with alternative substrates: lactate, acetate, citrate, glucose, galactose, glycine, or tryptone (10 mM). Sodium thiosulfate (10 mM), sodium sulfate (0 to10 mM), elemental sulfur (1%), yeast extract (0.2%) or cysteine (1 mM) were added to culture media to determine its ability to obtain energy from sulfate reduction and to use different sulfur sources. To test for arsenate resistance and the optimal concentration of As for energy metabolism, a range of concentrations between 0 and 16 mM was assayed. To find out if the growth of *Fas* on As(V) as electron acceptor was linked to oxidative phosphorylation and formation of proton or sodium gradients, growth was also evaluated with the addition of the protonophore 3,3′,4′,5-tetrachlorosalicylanilide (TCS, 20 μM) or the sodium-specific ionophore N,N,N′,N′-tetracyclohexyl-1,2-phenylenedioxydiacetamide (ETH2120, 20 μM; Tremblay et al., 2012; Wang et al., 2016).

### Analytical methods

To evaluate the arsenate and sulfate reducing activity, arsenic concentrations in the culture medium from bacterial cultures were measured after filtering through 0.02 μm pore size using Hydride Generation Atomic Absorption Spectroscopy (HG-AAS) and the As speciation, As(III) and As(V), was analyzed using a Chromatography PSA 10.055 Millennium Excalibur. Lactate, acetate, and sulfate were quantified by ion chromatography (Dionex ™ 3,200, Thermo Scientific) with an IonPac™ AS11-HC (4×250) analytical column with AG11-HC pre-column, injected with 10 μl of filtered bacterial culture supernatant, using a run time of 15 min at 30°C and a flow of 1.5 ml min^−1^. To calculate the concentrations according to the peak area, linear standard curves were performed in the range of 0–100 mg L^−1^ (R^2^˃0.99).

### Trx and TR enzymatic assays

To evaluate the involvement of the Trx system in the early (30 min) and late (8 h) response to As(V) exposure, the Trx and TR activities were measured at 30°C using 50 μg of whole cell extracts as described previously (Norambuena et al., 2012), a control test without exposure to As(V) and one more without cell extract were included in the experiment. Total Trx activity was determined by the insulin precipitation assay (Holmgren, 1979). The standard assay mixture contained 0.1 M potassium phosphate (pH 7.0), 1 mM EDTA, and 0.13 mM bovine insulin in the absence or in presence of the cellular extract, the reaction was started upon the addition of 1 mM DTT and the increase of the absorbance at 650 nm produced by the reduction of the insulin alpha-chain was monitored. TR was assayed for reductive activity toward 5,5-dithio-bis-(2-nitrobenzoic acid; DTNB) with NADPH to form 5-thio-2-nitrobenzoic acid (TNB), producing a strong yellow color that was measured at 412 nm (Arner et al., 1999).

### DNA purification

Bacterial DNA was extracted and purified using the High Pure PCR Template Preparation kit (Roche, cat. n° 11,796,828,001) according to the manufacturer’s protocol. PCR products were purified using the QIAquick PCR Purification kit (QIAGEN, cat. n° 28,104), and digested DNA products were extracted from the agarose gel using the QIAquick Gel Extraction kit (QIAGEN, cat. n° 28,704), according to the manufacturer’s instructions.

### PCR conditions

The presence of an *arrA* gene in the *Fas* genome was assessed by a PCR assay performed using the universal *arrA*f and *arrA*r primers to target an *arrA* internal ∼160–200 bp DNA fragment, as previously described (Malasarn et al., 2004). Genomic DNA from *Shewanella* sp. strain ANA-3 was used as a positive control. To clone the *arsC*-1*_Fas_* and *arsC*-2*_Fas_* genes, specific primers were designed based on both nucleotide sequences obtained from *Fas* genome and modified to include *Xho*I and *Hind*III restriction sites (Supplementary Table S1). The PCR assays were performed with the Phusion High-Fidelity DNA Polymerase (Thermo Scientific, F-350S) according to the manufacturer’s instructions. The following PCR conditions were used: initial denaturation at 95°C for 30 s, followed by 30 cycles of 98°C for 10 s, 56.1°C (*arsC*-1*_Fas_*) or 60.0°C (*arsC*-2*_Fas_*) for 25 s, and 56.1°C (*arsC*-1*_Fas_*) or 60.0°C (*arsC*-2*_Fas_*) for 1 min, and a final elongation at 56.1°C or 60°C for 3 min. Then, PCR products were visualized by electrophoresis and purified from the 1.5% agarose gel. Screening for recombinant colonies was made by PCR as previously described (Green and Sambrook, 2019) using the PCR conditions described above and the GoTag® kit (Promega, M3001).

### Cloning, heterologous expression of *arsC* genes, and evaluation of As(V) resistance

To confirm if *Fas* ArsC confers resistance to As(V), the *arsC*-1*_Fas_* and *arsC-*2*_Fas_* genes were amplified as described above. The purified PCR products were subjected to A-tailing with Taq DNA polymerase and to ligation into a T-vector (pGEM®-T Easy Vector, Promega, cat. n° A1360). The ligation product was used to transform *E. coli* JM109 (Competent Cells, Promega, cat. n° L2005) and recombinant clones were checked by sequencing. Plasmids with the *arsC*-1*_Fas_* and *arsC-*2*_Fas_* correct sequences were purified by miniprep (Wizard® Plus SV Minipreps, Promega, cat. n° A1360) and double digested with *Xho*I and *Hind*III (Thermo Scientific™, cat. n° ER0691 and ER0501, respectively) to release the inserts, which were subsequently purified and ligated upstream the His-tag into the expression vector pTrcHis2 (Invitrogen™, cat. n° V36520). The recombinant vectors were transformed into the *E. coli* WC3110 Δ*arsC* strain. Positive clones were cultured in LB medium with ampicillin 50 μg/ml for 12 h at 37°C. The expression of both *arsC* genes was induced with IPTG 1 mM and the conferred ability to growth in the presence of 0, 0.5, 1, 1.5, 2.5 and 5 mM arsenate was monitored by OD_600_ and compared to a clone of *E. coli* WC3110 transformed with the pTrc-*lacZ* used as control. The specific growth rate (μ) was calculated with the equation: μ = (lnX-lnX_0_)/(t-t_0_), where X and X_0_ represent the cell concentration or OD_600_, and t and t_0_ the time. The doubling time (t_d_) was determined using the equation: t_d_ = ln2/μ.

### Bioinformatic analysis

The genome of *Fas* was sequenced on an Illumina MiSeq platform at MR DNA (Molecular Research LP, Shallowater, TX, United States) as described in Serrano et al., 2017. Sequencing the library obtained with Nextera DNA Sample Preparation Kit, Illumina, an estimate of 20,000 (2 × 300-bp paired-end) reads with >50-fold coverage was retrieved. A draft genome of ~5.1 Mbp was assembled *de novo* using Newbler v2.0.01.14 and contained 57 contigs with 4,780 genes identified using RAST (Brettin et al., 2015). The genomes of *Fusibacter* sp. strain 3D3 (BDHH00000000.1), *F. ferrireducens* strain Q10-2^T^ (JADKNH000000000.1), *F. paucivorans* strain SEBR 4211^T^ (JAHBCL000000000.1), *F. tunisiensis* strain BELH1^T^ (JAFBDT000000000.1), *Fusibacter* sp. strain A1 (JABKBY000000000.1) were obtained from the NCBI database and also annotated on the RAST platform using default settings.

The genome sequence of *Fas* was screened to search for genes encoding components and regulators of arsenic redox and transport system, as well as those associated with thiol redox systems and energy metabolisms (Serrano et al., 2017). Curation of genes of interest was performed by reciprocal analysis against each other and the sequences available in the public databases to establish similarities and differences regarding gene identity, structure and function, gene context and control signals (Altschul and Lipman, 1990; Altschul et al., 1997; Sigrist et al., 2002; Tatusov et al., 2003; Punta et al., 2012). The sequence collections available on the NCBI (Benson et al., 2009) and the Comprehensive Microbial Resource of the J. Craig Venter Institute (Rockville, MD, United States; Davidsen et al., 2010) websites facilitated comparative genomic studies with other organisms of interest whose genome sequencing had already been completed.

For the classification of the *Fas* ArsC into families, depending on different thiol-disulfide relay mechanisms, its sequences were compared with known and putative arsenate reductases dependent of Grx or Trx proteins from *E. coli* (P0AB96), *Staphylococcus aureus* (P0A006), *Fusibacter* sp. 3D3 (WP_069871881, ArsC-1 and WP_069871901, ArsC-2) and *Citrobacter* sp. TSA-1 (PAX80297; PAX80313 and PAX80519). The protein subcellular localization was predicted with PSORTb v3.0.2.^1^ To determine the presence of transmembrane regions, DeepTMHMM^2^ was used.

For the verification of the EtfB (electron transfer flavoprotein subunit beta) domain conservation across *Fusibacter*, previously known proteins from *Geobacter metallireducens* GS-15, *Thermotoga maritima* MSB8, *Clostridium ljungdahlii* PETC, *Rhodopseudomonas palustris* BisA53, *Acetobacterium woodii* WB1, *Clostridium kluyveri* DSM 555, *Acidaminococcus fermentans* VR4, and *Megasphaera elsdenii* T81 were used (Garcia Costas et al., 2017). The protein sequences were obtained from the NCBI database, aligned by MUSCLE using the default parameters (Edgar, 2004) and visualized by CLC Genomic Workbench 8.5.1 (Qiagen). Subsequently, using BLAST on the RAST platform, EtfB-type proteins were searched in *Fusibacter* genomes, and the results were verified by BLASTp in the NCBI database. A similar procedure was applied to the products of the remaining genes.

The 16S similarity was determined with BLAST Global Alignment using the sequences from *Fusibacter* sp. strain 3D3 (GCA_001748365), *F. paucivorans* (NR_024886), *F. tunisiensis* (GCA_016908355), *F. bizertensis* (KJ420408), and *F. fontis* (LM999901).

## Results

### Microbial growth

**Figure 1:**
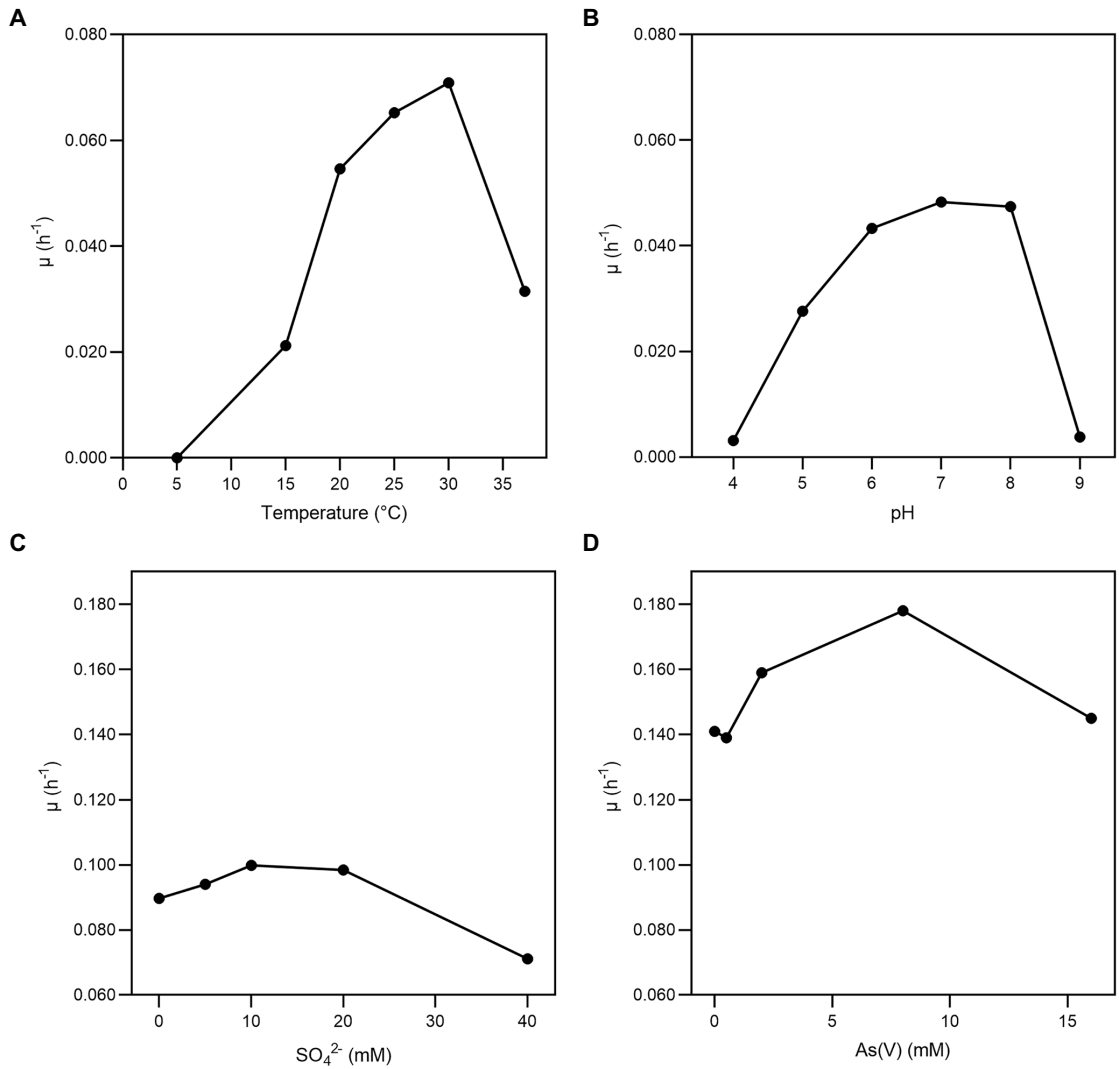
Growth of *Fas* in arsenate-containing medium. Specific growth rate (μ) of *Fas* growth on increasing temperatures **(A)**, pH **(B)**, concentrations of SO_4_^2−^ **(C)**, and As(V) **(D)**. Optimum temperature (30°C), pH (7), and concentrations of As (2 mM) and sulfate (20 mM) were used in the experiments except when different values of the corresponding variables were analyzed.

**Figure 2:**
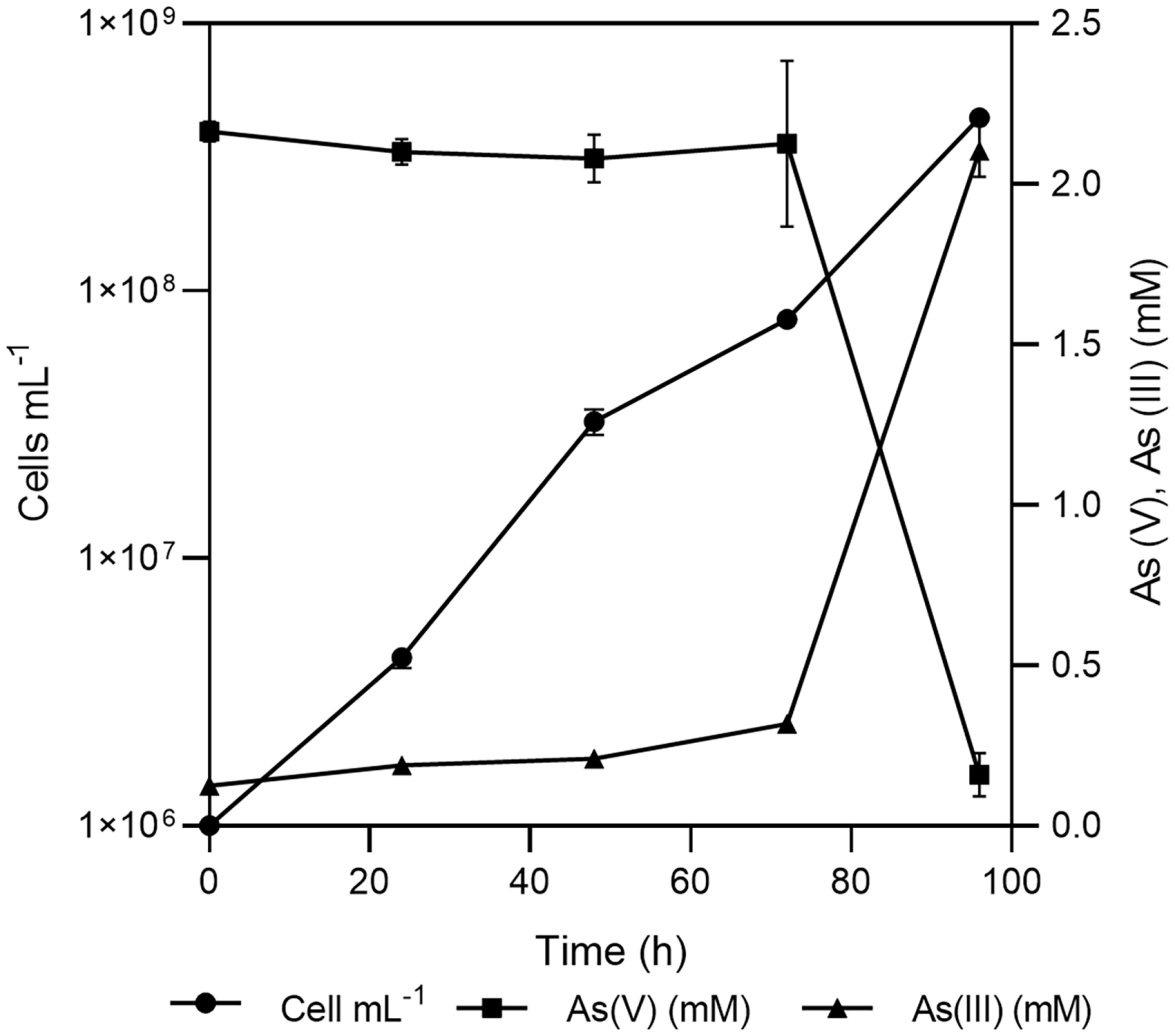
Growth of *Fas* in Newman-modified minimal medium with lactate (10 mM), sulfate (20 mM), arsenate (2 mM), yeast extract (0.1%), NaCl (10 g L-1), and cysteine (1 mM). Error bars represent the standard error of the mean of triplicate cultures. Sterile control experiments were also performed but the results were not shown for clarity.

*Fas* grew optimally at temperatures ranging from 20 to 37°C, with the lower doubling time (t_d_) observed at 30°C (Figure 1A) and under neutral (6–8) pH conditions (Figure 1B). *Fas* grew in synthetic medium containing up to 50 g L^−1^ of NaCl, and the optimum was 10 g L^−1^ (Serrano et al., 2017). No growth differences were detected at increasing SO_4_^−2^ concentrations (Figure 1C). The highest specific growth rate (μ) was observed at 2–8 mM of As(V) and pH 7 in anaerobiosis, but it grew in the range of 0.5 to up to 16 mM of As(V; Figure 1D). The lowest concentration of As(V) that completely prevented growth (MIC) of *Fas* was 24 mM. Liquid cultures reached total bacterial numbers between 3.1×10^8^ and 6.5×10^8^ cells mL^−1^ at the stationary phase (Figure 2).

No significant changes were noticed in the doubling time in minimal medium plus arsenate (2 mM) by the addition of sulfate (0 to 20 mM; Figure 1C; Serrano et al., 2017). *Fas* grew with a doubling time of 0.35 h in minimal medium plus arsenate (2 mM) and sulfate (20 mM) as electron acceptors, which increased to 4 h when not supplemented with arsenate (Figure 1D).

### Features of *Fusibacter* sp. strain 3D3 metabolism

#### Substrates and products

The strain was not able to grow in aerobiosis but grew in anaerobiosis on lactate in the presence of sulfate and arsenate as electron acceptors. Defined as a heterotrophic strain, *Fas* could use lactate, glucose and tryptone and required yeast extract to grow (Table 1). Growth without the addition of electron acceptors was successful up to the second subculture (data not shown) as it was in the previously reported culture medium for *Fusibacter* (Ravot et al., 1999). Besides, the highest As(V) to As(III) reduction ratio was evidenced between 72 and 96 h (Supplementary Figure S1).

**Table 1:** Comparison of the characteristics of *Fusibacter* sp. strain 3D3 and other *Fusibacter* type strains from literature, with sequenced genomes.

Characteristics	*Fas*	1	2	3	4	5
*Morphology*	Spindle-shaped rod	Rod	Spindle-shaped rod	Rod	Rod	Rod
*Temperature for growth (°C)*						
Range	20–35	15–40	20–45	15–45	15–35	8–45
Optimum	30	30	37	30	30	32
*pH for growth*						
Range	5.0–9.0	5.8–8.4	5.7–8.0	5.5–8.5	5.5–8.2	7.0–10.5
Optimum	7	7	7.3	7	7.2	8.5
*NaCl concentration for growth (g L^-1^)*						
Range	0–50	0–100	0–100	0–35	0–50	0–60
Optimum	2–8	30	0–30	1	5	30
*As(V) concentration for growth (mM)*						
Range	0–16	n.a	n.a	n.a	n.a	n.a
Optimum	2–8	n.a	n.a	n.a	n.a	n.a
*Electron acceptor utilized*						
Thiosulfate	+	+	+	−	+	+
Elemental sulfur	+	+	+	+	+	+
Sulphate	+	−	−	−	−	+
*DNA G + C content (mol%)*	37.6	43	38.2	37.6	37.4	37.4
*Substrates utilized*						
Lactate	+	−	−	−	−	n.a
Acetate	−	−	−	−	−	n.a
Citrate	−	n.a	n.a	n.a	n.a	n.a
Glucose	+	+	+	+	+	+
Galactose	−	−	−	+	+	+
Glycine	−	n.a	n.a	n.a	n.a	n.a
Tryptone	+	n.a	n.a	n.a	n.a	n.a
Cellobiose	n.a	−	+	+	+	−
Fructose	n.a	−	+	−	+	+
Maltose	n.a	+	−	+	+	+
Ribose	n.a	−	+	+	+	−
Sucrose	n.a	+	−	+	+	+
Trehalose	n.a	+	−	−	+	+

*Fas*: *Fusibacter* sp. strain 3D3 (Serrano et al., 2017); 1: *F. paucivorans* strain SEBR 4211^T^ (Ravot et al., 1999); 2: *F. tunisiensis* strain BELH1^T^ (Ben Hania et al., 2012); 3: *F. fontis* strain KhalAKB1^T^ (Fadhlaoui et al., 2015); 4: *F. bizertensis* strain LTF Kr01^T^ (Smii et al., 2015), and 5: *F. ferrireducens* strain Q10-2^T^ (Qiu et al., 2021). n.a., not analyzed.

*Fas* can be differentiated from *F. paucivorans*, *F. tunisiensis*, *F. fontis*, *F. bizertensis* and *F. ferrireducens* by its use of lactate as substrate, of sulfate as electron acceptor, the NaCl concentration for growth, its genomic DNA G + C content (Table 1) and its phylogeny (Qiu et al., 2021). Concerning the resistance to arsenic, to our knowledge it was not reported for other isolated members of the genus.

Lactate was consumed (12.3 mM, with 0.08 mM of acetate, and 3 mM of butyrate formed) while arsenate (1.85 mM) and sulfate (2.3 mM) were reduced. The amount of arsenite formed could not be determined quantitatively, as it tended to precipitate as yellow arsenic sulfide. On the other hand, sulfate reduction by *Fas* was demonstrated by sulfide and arsenic sulfide mineral production. Interestingly, neither sulfate nor thiosulfate reduction was involved in energy conservation as it has been reported for other members of the *Fusibacter* genus (Ravot et al., 1999; Ben Hania et al., 2012).

The ability of *Fas* to use lactate as electron donor when reducing arsenate suggests that arsenate respiration supports its growth. However, the arsenate reduced/lactate oxidized molar ratio observed was 0.32 ± 0.044 and the acetate produced/lactate consumed ratio was 0.23 ± 0.05, when the theoretical values predicted for isolated arsenate reduction reactions when lactate is transformed to acetate by respiring microorganisms are 2 and 1, respectively (Macy et al., 2000). In addition, the concomitant reduction of sulfate and the production of an arsenic sulfide precipitate does not allow an accurate quantification of arsenite and sulfide during *Fas* growth (Serrano et al., 2017).

#### Assessment of specific sulfur species source for growth

The lack of differences in the sodium sulfate dose curve led us to study the role of sulfur sources in arsenate reduction. *Fas* cultures with sodium sulfate, sodium thiosulfate and elemental sulfur were performed and combined with organic sulfur such as yeast extract and cysteine, both supplements required in Newman’s medium (Supplementary Figure S1). Culture without any source of inorganic sulfur was also carried out. All cultures were performed with 2 mM As(V).

The behavior of *Fas* cultures amended with sodium sulfate and sodium thiosulfate did not show significant differences, reaching the highest level of arsenate reduction with Yeast/Cys complete medium. Furthermore, the intake of cysteine (empty square) as unique source of organic sulfur appeared to rise up to 50% of the total arsenate reduction in all conditions at 96 h, and it was especially evident when inorganic sulfur was absent. Cultures supplemented only with yeast extract (filled triangle) induced lower arsenate reduction ratio than cysteine in all experiments. Growth (cell number) and sulfide production were also measured (Supplementary Figure S1).

#### Assessment of the role of proton/sodium gradient

**Figure 3:**
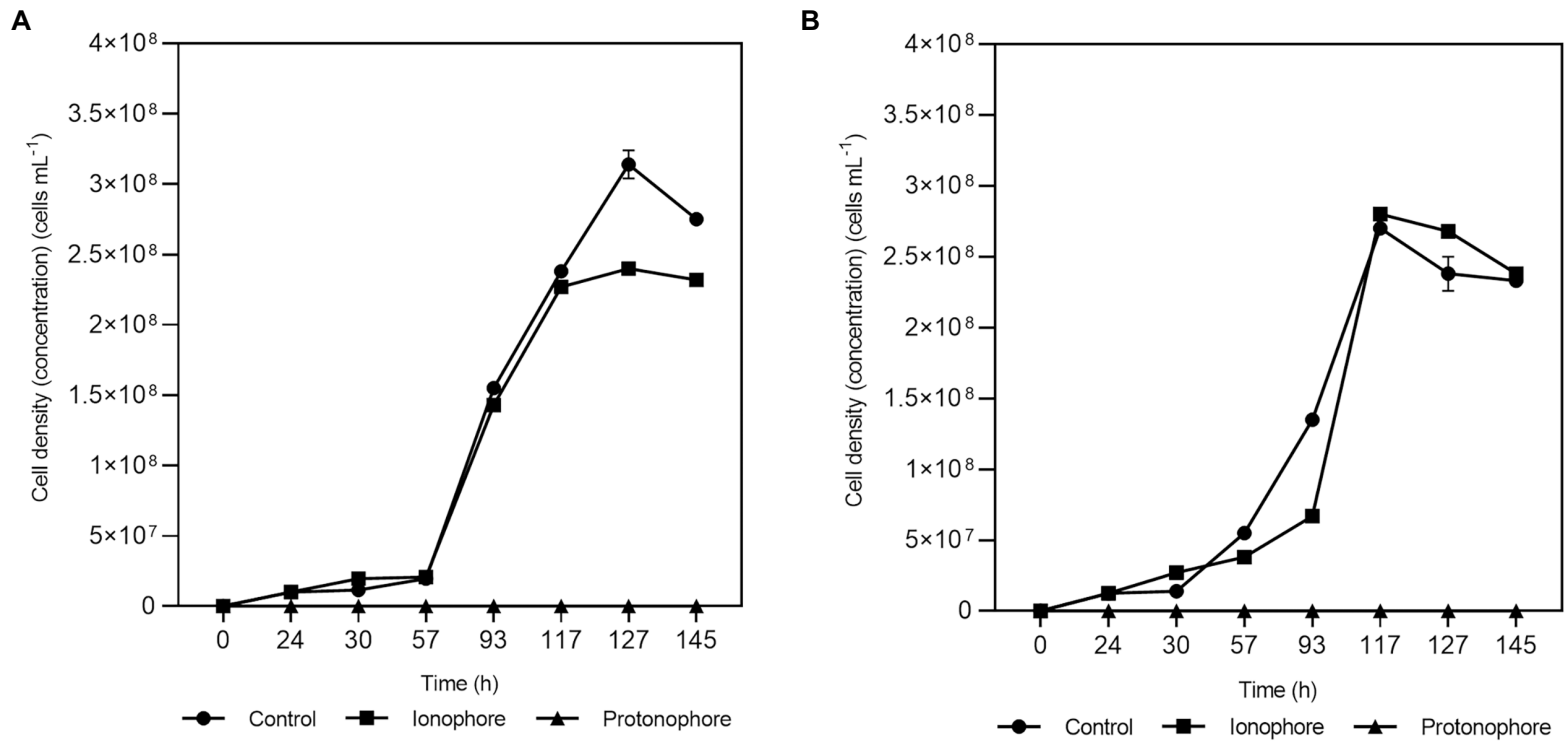
Effects of ETH2120 and TCS on *Fas* growth. Growth curves with lactate-sulfate-arsenate **(A)** and lactate-arsenate **(B)**, with addition of 20 μM ETH2120 as ionophore (■), 20 μM TCS as protonophore (▲) or no addition (●). Error bars show standard deviation of duplicates.

The addition of 20 μM of sodium ion ionophore ETH2120 did not have a significant influence on the growth of the strain with lactate as the electron donor whether sulfate-arsenate (Figure 3A squares) or only arsenate (Figure 3B squares) were present as electron acceptors. On the other hand, 20 μM of the protonophore TCS completely inhibited the growth on lactate-sulfate-arsenate (Figure 3A triangles) and lactate-arsenate (Figure 3B triangles). Growth experiments showed that lactate-sulfate-arsenate and lactate-arsenate were insensitive to the Na^+^ ionophore ETH2120 but were highly sensitive to the protonophore TCS.

In the protonophore test, the resting cells were also decreasing and demonstrated to be highly sensitive to TCS suggesting that *Fas* needed the proton gradient for energy generation. Moreover, the inability of the strain to grow in the presence of TCS is consistent with the role of that gradient in the generation of a proton motive force.

#### Assessment of the Trx system in the response to arsenate exposure

**Figure 4:**
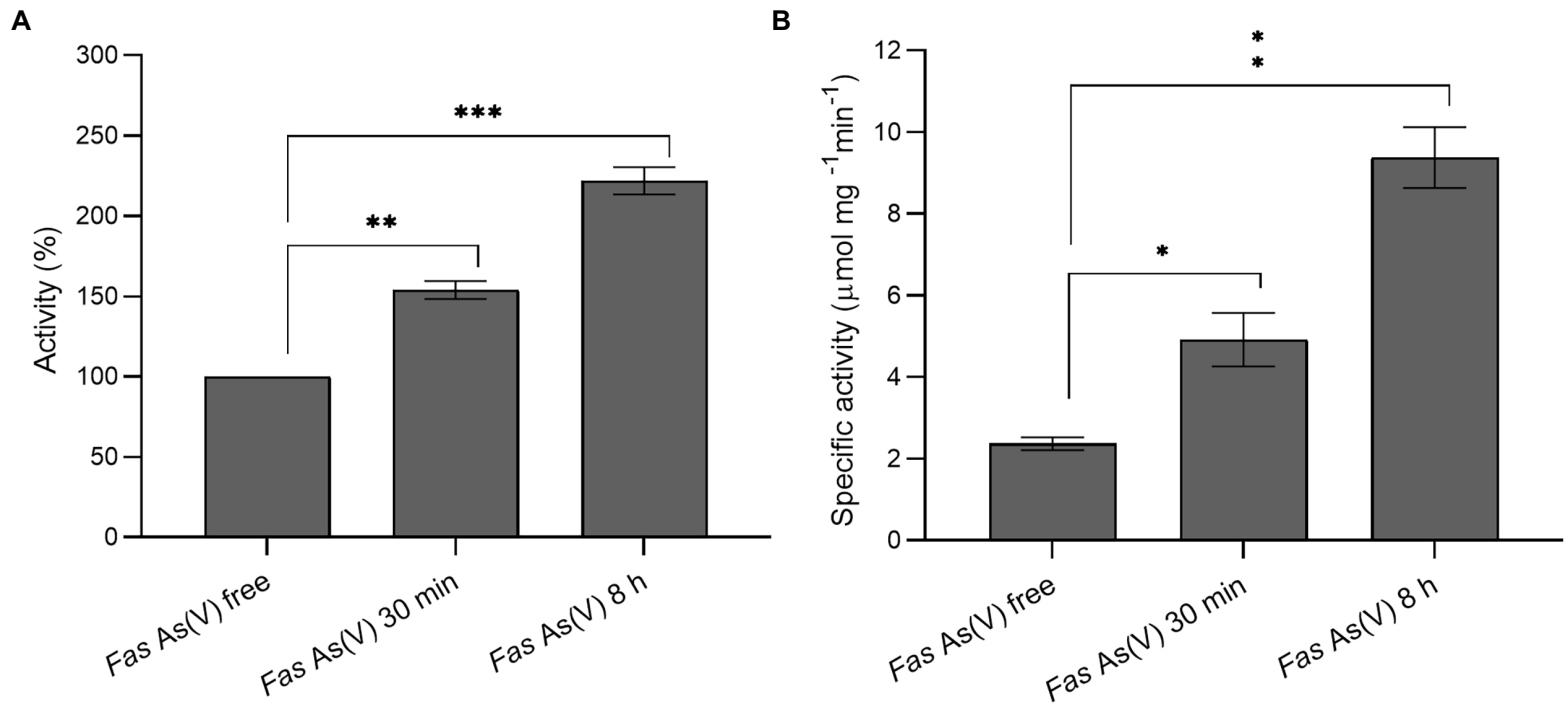
Measurement of thioredoxin and thioredoxin reductase activities. **(A)** Thioredoxin activity of *Fas* cells exposed to As(V) compared to the activity of cells grown without As(V). **(B)** Specific thioredoxin reductase activity of *Fas* before and after As(V) exposure. *p < 0.05; **p < 0.01; ***p < 0.001.

To gain insight into the thiol redox system involved in arsenate reduction and considering that the reductase ArsC of *Fas* was inferred by homology to be from the Trx/TR-dependent class, Trx (Figure 4A) and TR (Figure 4B) activity analysis were performed with cellular extracts. In addition, we compared the Trx activity with representative of Gram-positive (*B. subtilis*) and Gram-negative (*E. coli*) bacteria. The activity of Trx and TR increased after the exposure to arsenate (Figure 4). The Trx enzymatic activity was more than 7-fold higher than that determined in *E. coli* ATTC 4468 and *B. subtilis* HB 7038 without As(V; data not shown). A significant increase (*p* < 0.01) of Trx at 30 min (1.5-fold) and 8 h (2.3-fold) of As(V) exposure, and TR activities (2 and 4-fold at the same times) were observed. The results evidenced that Trx and TR were induced after the As(V) exposure.

### Genomic features

#### Genes involved in arsenate reduction and energy metabolisms

As noted in Tables 2, 3 and Supplementary Table S2, *Fas* draft genome sequence has genes coding for proteins involved in electron bifurcation and in arsenic, phosphate, and sulfur metabolisms. Arsenic detoxification genes (*arsACMR; acr3*) are clearly present in *Fas* genome (Serrano et al., 2017). *Fas* contains genes coding for two putative cytoplasmic arsenate reductases with only 32% of identity in their aminoacid sequences, both clustering with genes coding for the thioredoxin-coupled family (Supplementary Figure S2). We performed an *in silico* analysis of the *arsC*-1*_Fas_* and ArsC-2*_Fas_* sequences, together with other previously known/characterized ArsC proteins, to determine their possible cellular localization. The results of the analysis indicate that they are soluble proteins possibly located in the cytoplasm (Supplementary Table S2).The revisited genomic context of *arsC*-2 (*arsD* > *arsR*-2 > *pno* > *acr3* > *arsC*-2; Serrano et al., 2017) includes genes encoding for an arsenical resistant operon repressor (ArsD), a transcriptional regulator (ArsR), a 4Fe-4S ferredoxin (Pno, pyridine nucleotide-disulfide oxidoreductase NADH dehydrogenase), an arsenite efflux permease (Acr3), and the arsenate reductase (ArsC-2; Slyemi and Bonnefoy, 2012; Table 2). ATPase encoding gene that provide energy for arsenite efflux (*arsA*), included in the canonical *ars* operon of other Clostridiales, was also found in *Fas* even in another genomic context. Otherwise, the genomic context of *arsC*-1*_Fas_* revealed the presence of genes coding for ferredoxin and a redox-active disulfide protein (thioredoxin). Besides, two TR encoding genes were also identified in the *Fas* genome by the BLAST analysis (Table 3; Supplementary Table S3).

**Table 2:** BLAST results (UniProtKB reference proteomes+SwissProt databases) of predicted proteins related to arsenic resistance, phosphate, and sulfur metabolisms in *Fas*.

Subsystem	Protein	Functional role	NCBI	Closest protein homology		
				Species	UniProt	*E*-value
Anaerobic reductases	AprB	Adenylylsulfate reductase β-subunit/uncharacterized protein 4Fe-4S ferredoxin	WP_084389230	*Roseburia* sp. CAG:100	R7R6L1	4 × 10^−25^
Arsenic Resistance	ArsR-1	Arsenical resistance operon repressor	WP_069871038	*Dehalobacter* sp. DCA	K4LCR7	2 × 10^−43^
	ArsR-2	Arsenical resistance operon repressor	WP_069871893	*Desulfitobacterium hafniense*	Q24NC4	3 × 10^−53^
	ArsD	Metalloregulator ArsR/SmtB family transcription factor	WP_06971895	*Acetoanaerobium sticklandii*	E3PWS8	4 × 10^−40^
	Pno	Pyridine nucleotide-disulfide oxidoreductase NADH dehydrogenase/Rhodanese homology domain	WP_069871897	*Anaerotalea alkaliphila*	A0A7X5HVA6	0
	ArsA	Arsenical pump-driving ATPase (EC 3.6.3.16)/Arsenite-activated ATPase ArsA	GAU79918	*Clostridium* sp. BNL1100	H2J8R6	2 × 10^−68^
	ArsC-1	Arsenate reductase	WP_069871881	*Geobacillus thermodenitrificans*	A4INR2	5 × 10^−29^
	ArsC-2	Arsenate reductase	WP_069871901	*Amphibacillus xylanus*	K0J2A1	2 × 10^−72^
	ArsM-1	S-adenosylmethionine-dependent methyltransferase	WP_069875650	*Alkaliphilus peptidifermentans*	A0A1G5AIU9	6 × 10^−69^
	ArsM-2	S-adenosylmethionine-dependent methyltransferase	WP_069876683	*Paenibacillus polymyxa*	E3E8M9	5 × 10^−91^
	Acr3	Arsenical-resistance protein	WP_069871899	*Clostridium sticklandii*	E3PWS9	0
	AoxS	Periplasmic sensor signal transduction his-kinase	WP_069876025	*Alkaliphilus oremlandii*	A8MKM5	0
	AoxR	Transcriptional regulator	WP_069876024	*Alkaliphilus oremlandii*	A8MKM4	0
Phosphate metabolism	Pit	Probable low-affinity inorganic phosphate transporter	WP_069871941	*Caldithrix abyssi*	H1XTK9	9 × 10^−137^
	Aqps/GlpF	Glycerol uptake facilitator	WP_084389148	*Bacillus subtilis*	P18156	1 × 10^−34^
	PstS	Phosphate-binding protein	WP_069873924	*Staphylococcus epidermidis*	Q5HPF2	1 × 10^−60^
	PstA	Phosphate transport system permease	WP_084388970	*Xylella fastidiosa*	Q87C89	6 × 10^−24^
	PstB	Phosphate import ATP-binding protein	GAU77660	*Clostridium sticklandii*	E3PWC5	2 × 10^−163^
	PstC	Phosphate transport system permease	GAU77658	*Desulfitobacterium dichloroeliminans*	L0F6E8	3 × 10^−171^

**Table 3:** BLAST results of predicted proteins related to electron bifurcation in *Fas*.

Subsystem	Protein	Functional role	NCBI	Closest Protein Homology		
				Species	UniProt	*E-value*
Electron Transport	RnfA	Electron transport complex protein RnfA	WP_069873490	*Acetobacterium woodii*	H6LC28	1 × 10^−91^
	RnfB	Electron transport complex protein RnfB	GAU77413	*Alkaliphilus metalliredigens*	A6TQH4	4 × 10^−160^
	RnfC	Electron transport complex protein RnfC	WP_069873483	*Acetobacterium woodii*	H6LC32	7 × 10^−158^
	RnfD	Electron transport complex protein RnfD	WP_069873485	*Acetobacterium woodii*	H6LC31	1 × 10^−118^
	RnfE	Electron transport complex protein RnfE	WP_069873489	*Acetobacterium woodii*	H6LC32	3 × 10^−92^
	RnfG	Electron transport complex protein RnfG	WP_069873487	*Acetobacterium woodii*	H6LC30	1 × 10^−48^
Oxidation–reduction process	Fdx-1	Ferredoxin	WP_069875417	*Anaerotignum neopropionicum*	A0A136WCN9	2 × 10^−63^
	Fdx-2	Ferredoxin	WP_069871884	*Anaerotignum neopropionicum*	A0A136WCN9	2 × 10^−47^
	Fdx-3	Ferredoxin	WP_069871041	*Sedimentibacter saalensis*	A0A562J5A8	1 × 10^−51^
	Trx-1	Thioredoxin reductase/ FAD/NAD-binding	WP_069873949	*Peptoclostridium acidaminophilum*	P50971	2 × 10^−138^
	Trx-2	Thioredoxin reductase/ FAD/NAD-binding	WP_069874932	*Youngiibacter fragilis*	V7I8R3	0
	AhpC-1	Alkyl hydroperoxide reductase subunit C	WP_069870906	*Pyrococcus horikoshii*	O58966	1 × 10^−84^
	AhpC-2	Alkyl hydroperoxide reductase subunit C	GAU76052	*Clostridium sticklandii*	E3PTE6	8 × 10^−113^
	NqrB*	Na(^+^)-translocating NADH-quinone reductase sub. B	GAU79379	*Finegoldia magna*	E1KXR0	5 × 10^−115^
Electron transfer flavoproteins	NfnA	NADH-dependent reduced ferredoxin:NADP oxidoreductase, α subunit	WP_069872221	*Thermotoga maritima*	Q9X1X4	1 × 10^−88^
	NfnB	NADH-dependent reduced ferredoxin:NADP oxidoreductase, β subunit	WP_069872546	*Escherichia coli* (strain K12)	P09832	2 × 10^−91^
	EtfA-2	Electron bifurcating butyryl-CoA dehydrogenase, α subunit	WP_069875593	*Ilyobacter polytropus*	E3HC30	1 × 10^−161^
	EtfB-2	Electron bifurcating butyryl-CoA dehydrogenase, β subunit	WP_069875592	*Maledivibacter halophilus*	A0A1T5K5G4	3 × 10^−141^
	Bcd	Electron bifurcating butyryl-CoA dehydrogenase (NAD^+^, ferredoxin)	WP_069875591	*Clostridium acetobutylicum*	P52042	0
	EtfA-1	Electron transfer flavoprotein, α subunit	WP_069871749	*Clostridium saccharobutylicum*	P53578	5 × 10^−128^
	EtfB-1	Electron transfer flavoprotein, β subunit	WP_069871747	*Clostridium amylolyticum*	A0A1M6NXL2	2 × 10^−133^
	LdhD	Lactate/Glycolate dehydrogenase, subunit LdhD/GlcD	WP_069871751	*Caldisalinibacter kiritimatiensis*	R1AW66	0
PFOR: pyruvate:ferredoxin oxidoreductase	PorA-1	Pyruvate synthase subunit PorA/Pyruvate oxidoreductase α chain	WP_069871797	*Thermotoga maritima*	O05651	2 × 10^−45^	PorA-2	Pyruvate:ferredoxin oxidoreductase, α subunit	WP_069874428	*Acidaminobacter hydrogenoformans*	A0A1G5RST4	6 × 10^−166^	PorB-1	Pyruvate synthase subunit PorB/Pyruvate oxidoreductase β chain	WP_175438347	*Thermotoga maritima*	Q56317	2 × 10^−93^
	PorB-2	Pyruvate:ferredoxin oxidoreductase, β subunit	WP_069874582	*Thermohalobacter berrensis*	A0A419T5M6	7 × 10^−135^

NCBI and UniProt denote the accession numbers. *The whole Nqr complex was included in Supplementary Table S3.

The dissimilatory arsenate reductase *arrAB* gene cluster, involved in anaerobic respiration using As(V) as electron acceptor, was not found in *Fas* as it has been previously reported (Serrano et al., 2017). However, several genes predicted to be involved in the synthesis of the molybdenum cofactor included in the known catalytic site of ArrA (Slyemi and Bonnefoy, 2012) and in other cytoplasmic iron–sulfur proteins that catalyze ferredoxin-dependent redox reactions (Buckel and Thauer, 2013) were identified in the genome of *Fas*.

The NADH-dependent reduced ferredoxin:NADP^+^ oxidoreductase, α and b subunits (NfnAB), evidenced by BLAST analysis against the *Pyrococcus furiosus* proteins (Lubner et al., 2017), is present in *Fas* and is conserved in the genomes of the *Fusibacter* genus. NfnAB is an electron bifurcating enzyme complex which couples the reduction of NADP^+^ with reduced ferredoxin (Fd_red_) and the reduction of NADP^+^ with NADH in a reversible reaction (Buckel and Thauer, 2018a).

Bacteria transport inorganic phosphate through the constitutive low-affinity Pit transporter (Phosphate inorganic transport), however it is prone to transport As(V), when present. The inducible high-affinity phosphate transport system Pst (Phosphate specific transport; Vera et al., 2008; Slyemi and Bonnefoy, 2012) solve this issue. Both systems are present in the *Fas* genome.

The transmembrane ATP synthases (F0F1-ATPase complex) which are involved in ATP synthesis by obtaining the energy of a transmembrane gradient created by the difference in protons (H^+^), and in ATP hydrolysis in the reverse direction reactions, are encoded in the *Fas* genome (Supplementary Table S4). The order is conserved in the genomes of the *Fusibacter* genus (subunits *I*, *A*, *C*, *C*, *B*, Delta, Alpha, Gamma, Beta, Epsilon).

In addition, the occurrence of the genes *rnfC*, *D*, *G*, *E*, *A*, *B* reported as encoding for the membrane-associated ferredoxin-dependent *Rhodobacter* nitrogen fixing (Rnf) complex was revealed by BLAST analysis in *Fas* genome, and in the whole genus. The Rnf complex is responsible for transmembrane Na^+^/H^+^ transport (Müller et al., 2008) and for Na^+^/H^+^ gradient harvesting (Biegel et al., 2011).

A search in *Fusibacter* genomes for genes involved in the fermentation process (Gutiérrez-Preciado et al., 2018) revealed the occurrence of genes coding for an aldehyde dehydrogenase and butanoate metabolism in most of them, while genes involved in lactate/pyruvate metabolism were not present neither in *F. tunisiensis* nor in *F. paucivorans*. Genes codifying for pyruvate decarboxylase, alcohol dehydrogenase (cytochrome c), and proteins involved in the citrate cycle were absent (Table 4).

**Table 4:** Presence of genes related to fermentative metabolism in *Fusibacter* genomes.

Metabolic Pathway	Gene function	Gene name	COG	KO	1	2	3	4	5
Glycolysis	Pyruvate decarboxylase	*pdc*	COG3961	K01568	No	No	No	No	No
	Alcohol dehydrogenase (cytochrome c)	*exaA*	COG4993	K00114	No	No	No	No	No
	Alcohol dehydrogenase (NADP^+^)	*AKR1A1*	COG0656	K00002	No	No	No	Yes	No
	Alcohol dehydrogenase	*eutG*	COG1454	K04022	Yes	Yes	No	Yes	Yes
	Aldehyde dehydrogenase (NAD^+^)	*ALDH*	COG1012	K00128	Yes	Yes	Yes	Yes	Yes
	Aldehyde dehydrogenase (NAD(P)^+^)	*ALDH3*	COG1012	K00129	Yes	Yes	Yes	Yes	No
	L-lactate dehydrogenase	*ldh*	COG0039	K00016	Yes	Yes	Yes	Yes	Yes
Pyruvate metabolism	D-lactate dehydrogenase (cytochrome)	*dld*	COG0277	K00102	Yes	Yes	No	No	Yes
	Formate C-acetyltransferase	*pflD*	COG1882	K00656	Yes	Yes	No	Yes	Yes
Butanoate metabolism	(R,R)-butanediol dehydrogenase/meso-butanediol dehydrogenase/diacetyl reductase	*butB*	COG1063	K00004	Yes	Yes	No	Yes	Yes
	Butyrate kinase	*buk*	COG3426	K00929	Yes	Yes	Yes	Yes	Yes
	Butyryl-CoA dehydrogenase	*bcd*	COG1960	K00248	Yes	Yes	Yes	Yes	Yes
Citrate cycle (TCA cycle)	Succinate dehydrogenase/fumarate reductase, flavoprotein subunit	*sdhA, frdA*	COG1053	K00239	No	No	No	No	No
	Succinate dehydrogenase/fumarate reductase, iron–sulfur subunit	*sdhB, frdB*	COG0479	K00240	No	No	No	No	No
	Succinate dehydrogenase/fumarate reductase, cytochrome b subunit	*sdhC, frdC*	COG2009	K00241	No	No	No	No	No
	Succinate dehydrogenase/fumarate reductase, membrane anchor subunit	*sdhD, frdD*	COG2142	K00242	No	No	No	No	No

1: *F. ferrireducens* strain Q10-2^T^; 2: *F. tunisiensis* strain BELH1^T^; 3: *F. paucivorans* strain SEBR 4211^T^; 4: *Fusibacter* sp. strain A1 (NCBI).

Searching for *etfB* genes in the *Fusibacter* genomes allowed us to find genomic contexts that would code for proteins involved in electron bifurcation. To verify the presence of key features (motifs 1 and 2) of the electron bifurcating EtfBs, an alignment of putative *Fusibacter* EtfBs and previously characterized proteins was performed (Supplementary Figure S3). We found that all *Fusibacter* genomes code for group 2A EtfBs, *Fas* and *F. ferrireducens* also code for group 2B, while only *F. paucivorans* code for group 2C elements.

**Figure 5:**
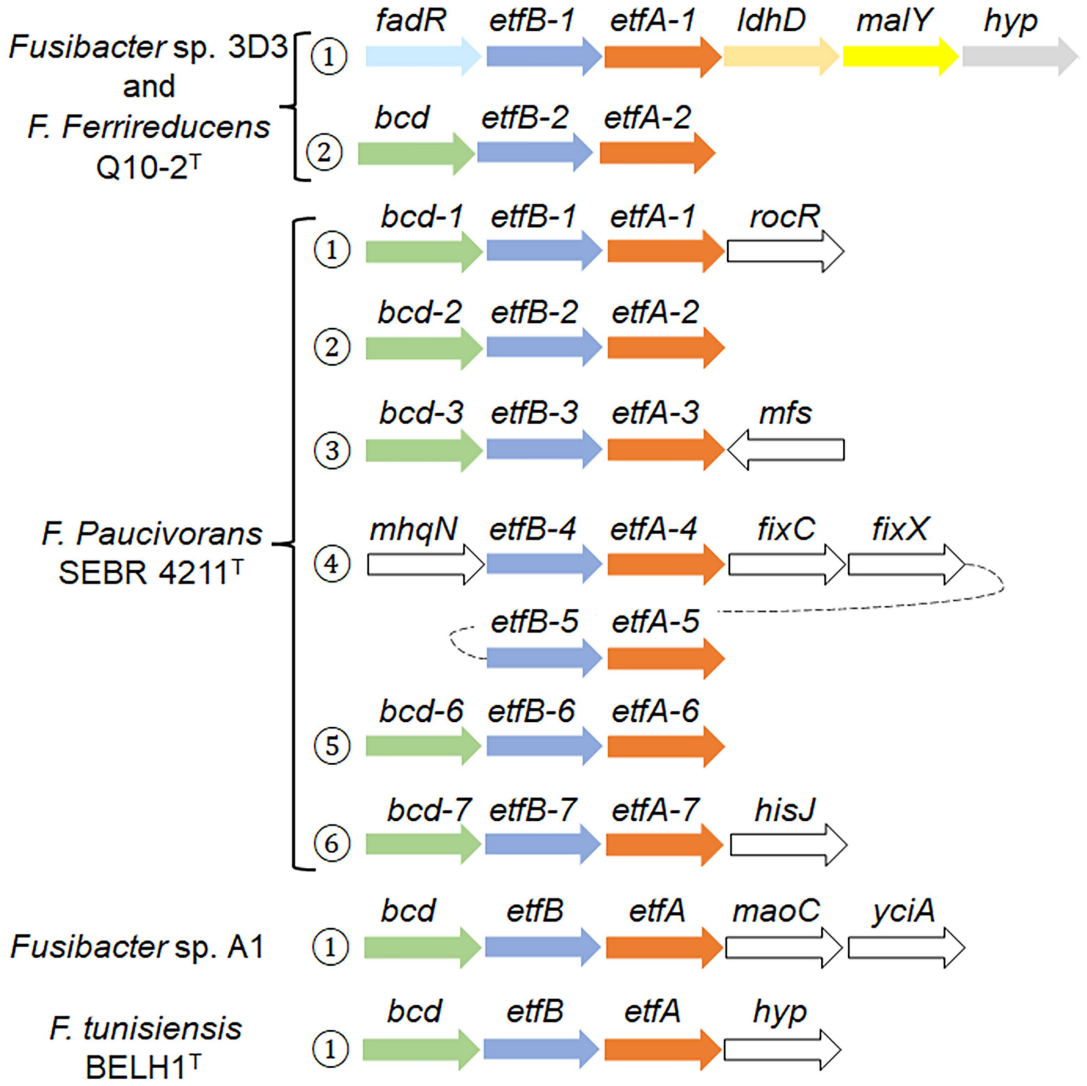
Genomic context of *etf* related genes in *Fusibacter*. Numbers in circles indicate the occurrence of the *etf* copies. The arrows represent the orientation of the gene (size is not at scale), and the same colors indicate homology, except white. The gene products are described in the text and the gene product accession numbers are listed in Supplementary Table S5.

To better understand their possible role in *Fas*, we compared the genomic contexts of the *etf* encoding genes in *Fusibacter* (Figure 5). All the analyzed genomes contain the gene encoding for the electron transfer flavoprotein subunit beta followed by the alpha subunit encoding gene. We found at least one copy of the genes encoding for EtfA, EtfB, and a putative butyryl-CoA dehydrogenase (Bcd) in *Fusibacter* genomes. Interestingly, *bcd* is always located upstream the *etf* genes cluster.

*Fas* and *F. ferrireducens* have two identical genetic arrangement. In context 1, *fadR* (which codifies for a transcriptional regulator) is located upstream *etfB-1* and *etfA-1* genes, and downstream of both are *ldh* that codifies for a lactate/glycolate dehydrogenase (COG0277), *malY* that codifies a putative pyridoxal 5′-phosphate (PLP)-dependent C-S lyase (COG1168) and a gene that codifies for a hypothetical protein conserved in both genomes, while in context 2, only a *bcd* gene was identified upstream *etfBA-2*.

Surprisingly, *F. paucivorans* contains seven copies of the *etfB-etfA* pair in six different contexts. Contexts 1, 2, 3, and 6 present the upstream arrangement with the *bcd* gene. In context 4, *mhqN*, which codifies for a nitroreductase family protein (cd02137), is found upstream *etfBA-4* while *fixC* and *fixX* are downstream and code for a flavoprotein dehydrogenase (COG0644) and a ferredoxin-like protein (COG2440) respectively. The fifth copy *etfBA-5* was identified downstream *fixX*. In addition, *F. paucivorans* has four orphan *etfB* genes, possibly belonging to the 2C2 group due to its phylogeny and because it does not have any *etfA* or *etfB* genes fused in a single open-reading frame, as it has been described in the group 2C1 (Garcia Costas et al., 2017). Independently, we also identified in *F. paucivorans* genes coding for a nitrogenase reductase and maturation protein (*nifH*), the regulatory proteins P-II (*glnA* and *glnB*) and α and β subunits of the nitrogenase (*nifD* and *nifK*). This opens the possibility that in this *Fusibacter* species some *etf* genes participate in nitrogen fixation. Indeed, no other *Fusibacter* possesses nitrogen fixation genes (results not shown).

*Fusibacter* sp. A1 and *F. tunisiensis* had only one specific genomic context with *etf*-related genes. In *Fusibacter* sp. A1, downstream *etfA* we found *maoC*, that codifies for an acyl dehydratase (COG2030), followed by *yciA*, coding for an acyl-CoA hydrolase (COG1607), both related to lipid transport and metabolism.

#### Detection of dissimilatory arsenate reductase *arrAB* genes

To corroborate the above mentioned, that the dissimilatory arsenate reductase *arrAB* gene cluster were not found in the draft genome sequence, an attempt was made to amplify these genes using specific primers, although an amplification product was not obtained either (Supplementary Figure S3). This suggests that a different mechanism, independent of ArrAB, is conferring the ability to obtain energy from arsenate reduction.

#### Heterologous expression of *arsC* genes

**Figure 6:**
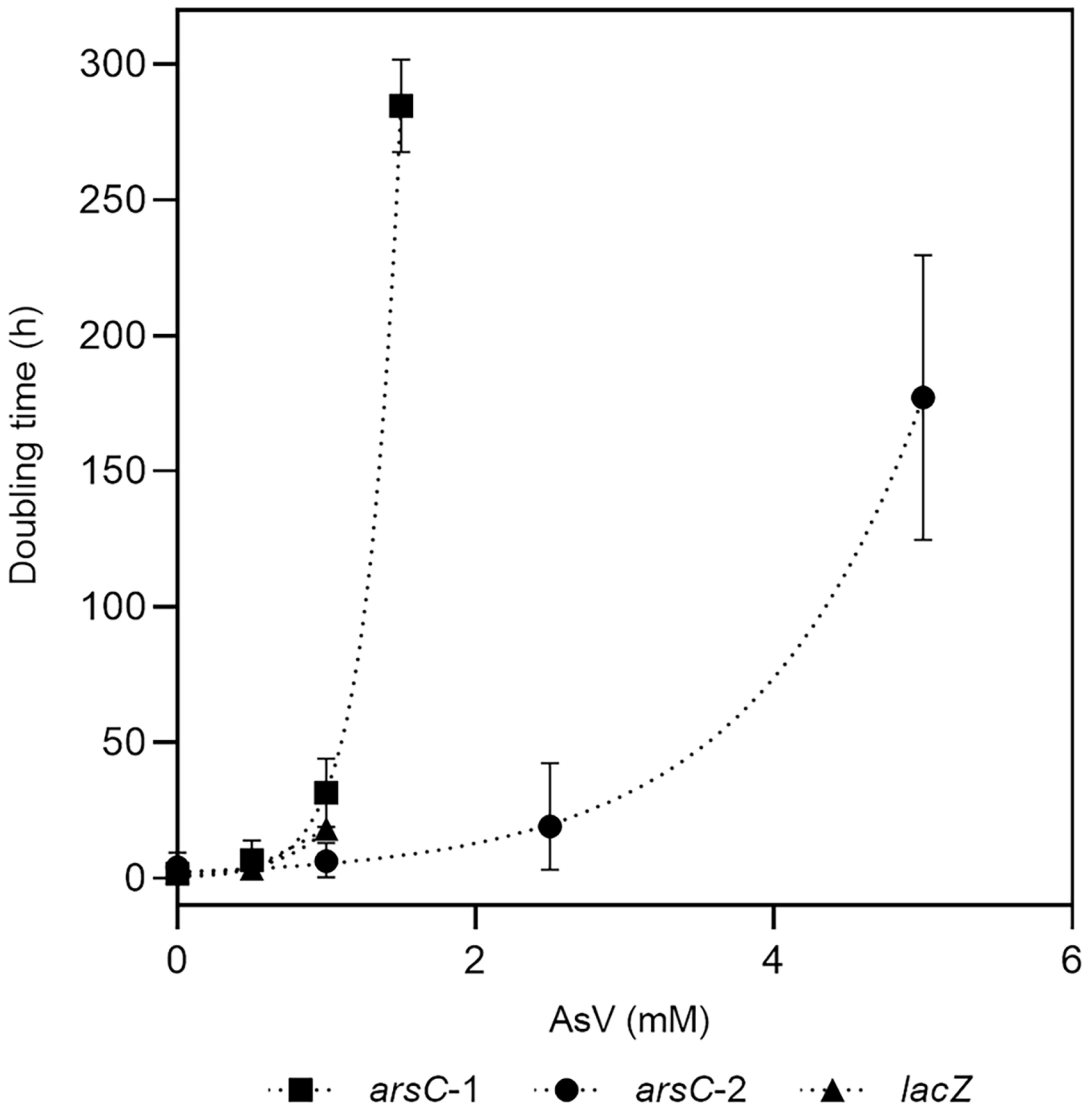
Evaluation of resistance to As(V) conferred by *arsC*-1 and *arsC*-2 from *Fas*. Growth of *E. coli* WC3110 Δ*arsC* strain complemented by *arsC*-1*_Fas_* SHT, *arsC*-2*_Fas_* SHT or *lacZ* genes in presence of As(V).

Two genes encoding arsenate reductases were identified in the *Fas* genome sequence and specific primers were designed for their amplification by PCR. The amplified genes (*arsC*-1*_Fas_* and *arsC*-2*_Fas_*; Supplementary Figure S4) were first cloned in the pGEM-T cloning vector, then released through enzymatic DNA digestion (Supplementary Figure S5) and ligated into the pTrcHis2A expression vector. The presence of the insert in the expression vector was checked by colony PCR (Supplementary Figure S6) or releasing the insert through DNA digestion of plasmids (Supplementary Figure S7). The activity of the gene product coded by the insert was tested by growing the recombinant *E. coli* WC3110 in the presence of As(V; Figure 6). Complementation of the *E. coli* WC3110 Δ*arsC* strain with the insert of both putative *arsC_Fas_* genes evidenced changes in As(V). A higher resistance to As(V) was conferred by ArsC-2*_Fas_* compared to ArsC-1*_Fas_*. The control strain *E. coli* WC3110 overexpressing *lacZ* did not grow at concentrations higher than 1 mM of As(V). Growth of *E. coli* WC3110 strain without insert was not observed. These biological data are the first metabolic evidence needed to confirm the existence of the proposed metabolism in *Fas*.

## Discussion

Phylogenetic analysis performed with the 16S rRNA genes had formerly grouped *Fas* inside the Gram-positive *Fusibacter* genus (Serrano et al., 2017). The comparison of the 16S rRNA sequences of *Fas* and other *Fusibacter* type strains revealed an identity of 98% with *F. ferrireducens*, 94% with *F. paucivorans*, *F. tunisiensis* and *F. fontis*, and 93% with *F. bizertensis*. Furthermore, The *in silico* average nucleotide identity (ANI) with its closest relative is 80.1% (Qiu et al., 2021). This gives us an additional indication that isolated 3D3 is a member of the *Fusibacter* genus.

Taking together the observed growth features of *Fas* compared with other species of the *Fusibacter* genus (Table 1) and the insights into their genome sequences (Tables 2, 3; Supplementary Table S1) we can propose a rationale to justify the singularity of the energetic metabolism of *Fas*. It grows strictly in anaerobiosis by reducing arsenate and using lactate as electron donor and its growth is improved by increasing arsenate concentration (Figure 1), being 2 mM the optimum level. To date, arsenic metabolism was not reported for the other *Fusibacter* species. Despite the arsenate reducing activity, the dissimilatory arsenate reductase *arrA* gene was not detected neither by the *Fas* genome sequence analysis (Serrano et al., 2017) nor by PCR assays (Supplementary Figure S3). Furthermore, neither *arrC* [which encodes the membranous subunit suggested to play the role of menaquinone oxidation and is present in some arsenate reducing bacteria (van Lis et al., 2013)], nor *omc* (encoding an outer-surface, octaheme c-type cytochrome), nor *cymA* genes (encoding a membrane-bound MKH2 oxidizing protein and reported in arsenate respiring *Shewanella* sp. strains; Murphy and Saltikov, 2007; Kim et al., 2011) were evidenced in the *Fas* genome (Table 2). The heterologous expression on *E. coli* WC3110 *ΔarsC* strain has allowed us to confirm that ArsC-1*_Fas_* and ArsC-2*_Fas_* are functional and confer arsenic resistance (Figure 6). Both *arsC_Fas_* genes belong to the Enterobacterial clade one (Zegers et al., 2001) and, therefore, encode a TR-dependent class of ArsC. In addition, the enzymatic analysis revealed a high Trx and TR activity in cells cultured with As, supporting the inference about the Trx dependence of the ArsC of *Fas*. As has been suggested before, Trx and TR provide early responses to oxidative stress in *Leptospirillum ferriphilum*. However, contrary to the observed in other microorganisms (Norambuena et al., 2012), a decrease in Trx activity was not observed in *Fas* after 8 h of exposure to As(V) probably because of the TR induction, that restores the Trx activity necessary for As(V) reduction. A 2.8-fold upregulation of Trx (Pandey et al., 2012) has been also observed in *Anabaena* after exposure to the oxidative stress generated by As (Pandey et al., 2012). Its ArsC enzyme has been classified, as well, inside the Trx dependent family (Pandey et al., 2013).

All the previously reported strains of the *Fusibacter* genus are fermentative bacteria (Ravot et al., 1999; Ben Hania et al., 2012; Fadhlaoui et al., 2015; Smii et al., 2015; Qiu et al., 2021). Interestingly, *Fas* can use lactate and glucose as substrates, while. *F. tunisiensis*, *F. paucivorans, F. bizertensis*, and *F. ferrireducens* cannot utilize lactate (Ravot et al., 1999; Ben Hania et al., 2012; Smii et al., 2015; Qiu et al., 2021). The genetic evidence agrees with the observed physiology on the culture conditions tested (Table 4). Furthermore, all the reported *Fusibacter* species have the ability to reduce sulfured nutriments (Ravot et al., 1999; Ben Hania et al., 2012; Fadhlaoui et al., 2015; Smii et al., 2015; Qiu et al., 2021). Sulfate reduction by *Fas* was demonstrated by sulfide and arsenic sulfide mineral production, and thiosulfate reduction was also positively checked. In addition, thiosulfate and sulfate were more efficient than sulfur to stimulate cell growth (Supplementary Figure S1) perhaps because of the low solubility of sulfur. Other *Fusibacter* species reduce thiosulfate and sulfur (but not sulfate or sulfite), and only *F. ferrireducens* shares with *Fas* the ability to reduce sulfate. Neither sulfate nor thiosulfate were involved in energy conservation in *Fas* as it has been reported for the other members of the *Fusibacter* genus (Ben Hania et al., 2012; Fadhlaoui et al., 2015). That feature could also be related to other cellular mechanisms present in microorganisms to cope with stress, such as arsenic stress, i.e., sulfur assimilation (Cleiss-Arnold et al., 2010). *F. paucivorans* growth experiments with sulfured nutriments revealed that the addition of thiosulfate relieved the inhibition produced by the H_2_ released by the glucose fermenting metabolism. In addition, a differential pattern of glucose fermentation products was observed in cultures with thiosulfate, represented by a decrease in butyrate levels together with an increase in acetate production (Ravot et al., 1999). As well as for other fermenting bacteria, those results confirm the Huber hypothesis that sulfur reduction plays a role of an electron sink reaction to prevent H_2_ accumulation from fermentation metabolism (Boileau et al., 2016). Therefore, the lactate/butyrate fermentation metabolism in *Fusibacter* should be regulated by the cellular redox state, resembling the reported for other *Firmicutes* (Detman et al., 2019). Interestingly, a redox-sensing transcriptional repressor gene encoding a protein whose DNA binding activity is modulated by the NADH/NAD^+^ ratio (Detman et al., 2019) is located downstream to the acetyl-CoA acetyltransferase encoding gene in *Fas* and in other *Fusibacter* genomes (data not shown). The acetyl-CoA acetyltransferase is in charge of the first step during the acetyl-CoA fermentation to butyrate pathway after the split in the three alternative fermentation pathways. Moreover, the results obtained from the growth experiments with and without addition of the protonophore TCS and the ionophore ETH2120 (Tremblay et al., 2012) revealed that *Fas* does require the formation of a proton gradient to get energy for growing on arsenate (Figure 3). In addition, the occurrence of genes encoding for the Rnf complex in *Fas* genome (Table 2) allows us to infer the capacity of *Fas* for energy conservation/utilization *via* proton translocating ferredoxin oxidation/reduction. Finally, the F0F1ATP synthase would couple ATP synthesis to the electrochemical gradient based on differences in the proton concentration generated.

In agreement with the genomic characterization of the ArsC*_Fas_* inside the Trx-dependent class, the enzymatic analysis has shown an increased level of Trx and TR activities after arsenate addition (Figure 4). Besides, it is known that thioredoxin is also involved in sulfur assimilation, evidenced in the early response to arsenic and in maintaining the cellular redox state (Cleiss-Arnold et al., 2010).

**Figure 7:**
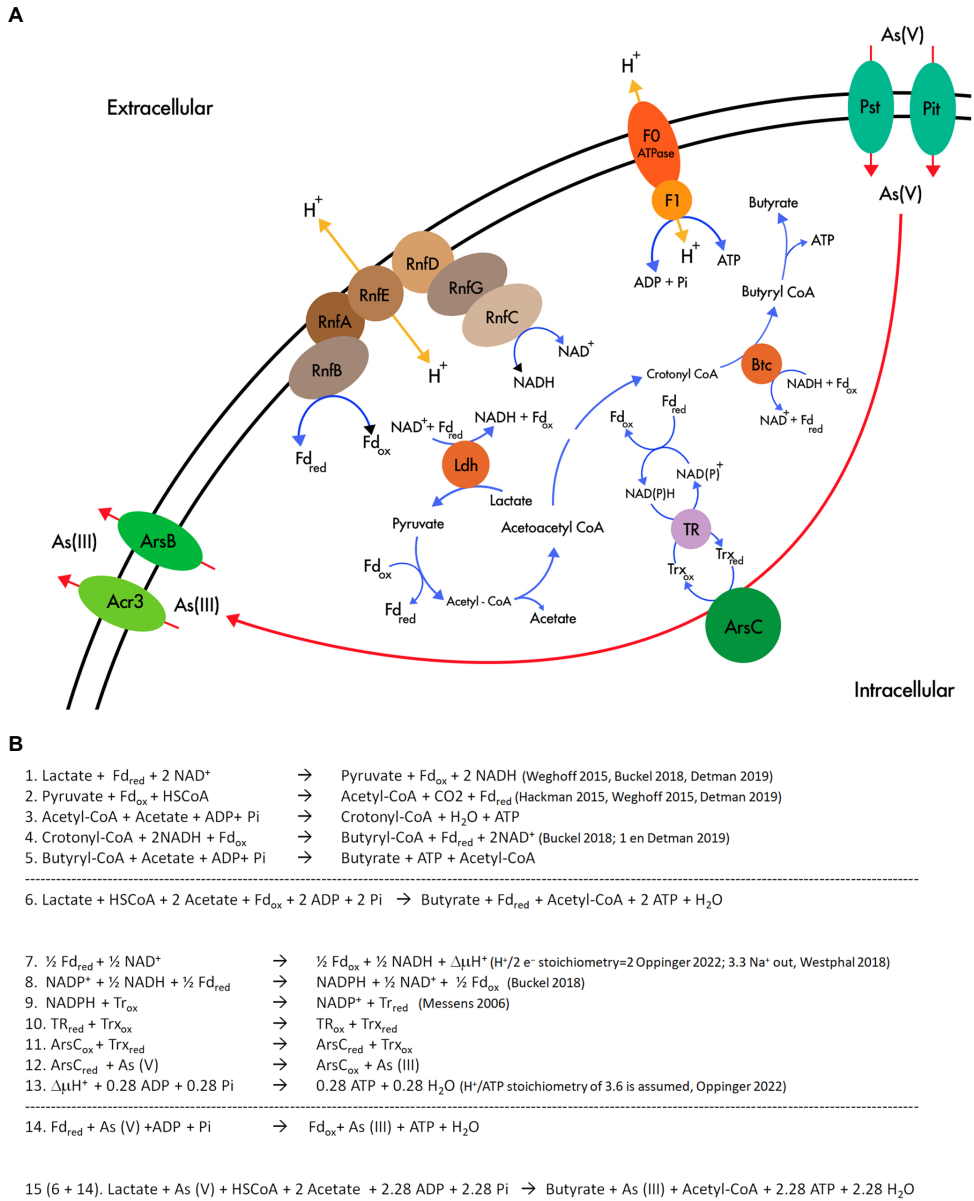
Flavin based electron bifurcation and its link with As(V) reduction. **(A)** Working model of the involvement of FBEB and the As(V) reduction in *Fas*. **(B)** Stoichiometry of the metabolism proposed in **(A)**.

*Fas* has all the known genomic resources for the pathway of lactate fermentation to acetate and butyrate in *Firmicutes* (Detman et al., 2019). Interestingly, the genomes of *Fas* and *F. ferrireducens* contain two genomic contexts that may be involved in the electron bifurcation process of the electron-transferring flavoproteins (EtfAB) type (Buckel and Thauer, 2018a). According to the model proposed for lactate and acetate transformation to butyrate (Detman et al., 2019), there must be a lactate dehydrogenase/EtfAB complex and a butyryl CoA dehydrogenase/EtfAB complex (Figure 7A). We propose that contexts 1 and 2 encode the elements for the transformation of lactate and butyryl CoA, respectively (Figure 5). Acetate production was observed in *Fas* and butyrate plus acetate production was confirmed in *F. paucivorans* (Ravot et al., 1999). *Clostridium butyricum* and *Acetobacterium woodii* were shown to transform lactate during fermentation by an enzyme complex of Ldh, EtfAB (Weghoff et al., 2015; Detman et al., 2019). Analysis of the genomes of *C. butyricum*, and *A. woodii* among others, showed conservation of the genetic context of at least the genes coding for these proteins (Weghoff et al., 2015;Poudel et al., 2018; Detman et al., 2019). Proteins related to butyryl CoA transformation involve butyryl-CoA dehydrogenase/electron transfer flavoproteins EtfA and EtfB (Li et al., 2008). This complex is also encoded in a conserved array (Poudel et al., 2018; Detman et al., 2019), like the genomic context 2 found in *Fas* (Figure 5). Furthermore, the occurrence of a NADH dependent reduced ferredoxin NADP^+^ oxidoreductase complex, the second type of FBEB complexes (Buckel and Thauer, 2018a), in *Fas* genome (Table 2) permits to hypothesize that energy for growth should be provided by the energetic link of cellular ferredoxin and NAD^+^ pools through the Rnf function to generate chemiosmotic potential when ferredoxin is higher than NADH level or, in reverse, for ferredoxin generation when NADH is higher (Westphal et al., 2018), for a more efficient metabolism in anoxic environments. In addition, Nfn could play the reported role of balancing the redox state of the pyridine nucleotide NAD(H) and NADP(H) pools and, in that way, favor the catabolic or anabolic reactions (Poudel et al., 2018). Also, the occurrence of multiple and different bifurcating (Bf) enzymes observed in *Fas* has been already detected in several *Firmicutes* genomes, and Bf-Ldh, Bf-Bcd and Nfn, that share NAD(H) and ferredoxin as common substrates, usually participate in those combinations (Lubner et al., 2017).

The revealed specialization for lactate fermentative metabolism present in *Fas* and already reported in *Firmicutes* (Detman et al., 2019), with the participation of FBEB and Rnf, supports the availability of NADH and Fd_red_ and ATP generation (Figure 7A and Equations 1–5 in Figure 7B). NADH and Fd_red_ should be the soluble electron carriers required for producing NADPH and starting the cascade of thiol reductases (NADPH→TR→Trx→ArsC; Messens and Silver, 2006) involved in As(V) reduction by ArsC Trx type of *Fas* (Figure 7A,B, equations 7–15). In that way, besides generating ΔμH^+^ coupled to ATP synthesis by ATP synthase, FBEB would conduct As(V) reduction by providing low-potential ferredoxin. The As(III) efflux pumps present in the Ars operon allow As(III) elimination and As_4_S_4_ precipitation outside the cells.

The analysis of the reported stoichiometry (Messens and Silver, 2006; Hackmann and Firkins, 2015; Weghoff et al., 2015; Westphal et al., 2018; Buckel and Thauer, 2018a) hints us that it is plausible that As(V) could play a role similar to that of CO_2_ in acetogenic bacteria (Buckel and Thauer, 2018b), of terminal acceptor of the electrons derived from reduced ferredoxin, the low potential electron carrier generated by electron bifurcation (Figure 7B).

This rationale allows us to formulate a hypothetical metabolism (Figure 7A) similar to the evidenced in other anaerobic microorganisms (Peters et al., 2018): Arsenate reduction provides additional energy to arsenate reducing fermenters independent of ArrAB for growing through a new mechanism that involves soluble ferredoxin electron carrier, FBEB complexes, the cytoplasmic ArsC, and the membrane-associated ion-translocating complex Rnf. As previously reported, this system could be regulated by the redox state (Detman et al., 2019). The energetic link of cellular NADH and ferredoxin should be the way in which the electrons reach As(V) in the cytoplasm, converting it in an electron sink/electron acceptor, similar to the role assigned to ferric iron in *F. ferrireducens* (Qiu et al., 2021).

Finally, the ecological relevance of the proposed metabolism was suggested by different approaches in two arsenic rich environments. A metagenomic analysis evidenced that the Trx-ArsC is much more diverse (assigned to Bacteroidetes, uncultured Proteobacteria, Firmicutes, Alphaproteobacteria, Deltaproteobacteria, Gammaproteobacteria, Actinobacteria, Verrucomicrobia, Spirochaetes, and Deinococcus-Thermus) than the Grx-ArsC (assigned only to Gammaproteobacteria) in the high altitude modern stromatolites at the base of the Socompa Volcano in the Argentinian Puna (Altiplano; Kurth et al., 2017). Furthermore, the predominant occurrence of genes predicted to encode Trx-ArsC instead of Grx-ArsC cytoplasmic arsenate reductase at As concentrations higher than 4 mg L^−1^ was evidenced by PCR amplification using three specific primer pairs for each type (Escudero et al., 2013). This work was done in a regional survey at the High Andes, the same environment where *Fas* was isolated from.

## Data availability statement

The datasets presented in this study can be found in online repositories. The names of the repository/repositories and accession number(s) can be found in the article/ Supplementary material.

## Author contributions

CD and MA conceptualized this study, designed the methodology, and conducted the research and formal data analysis. SM, NG, and SV contributed to the analysis and interpretation of the data. CD and MA wrote the first draft. All authors contributed to the review, editing, and revision of the article and approved the submitted version.

## Funding

This article was funded by FONDECYT Project 1100795 from the Chilean National Commission for Science and Technology (CONICYT), FIC-R 2015/BIP 40013423-0 and the Research Support from Minera Escondida Ltda. Project 32002137.

## Conflict of interest

The authors declare that the research was conducted in the absence of any commercial or financial relationships that could be construed as a potential conflict of interest.

## Publisher’s note

All claims expressed in this article are solely those of the authors and do not necessarily represent those of their affiliated organizations, or those of the publisher, the editors and the reviewers. Any product that may be evaluated in this article, or claim that may be made by its manufacturer, is not guaranteed or endorsed by the publisher.

## Footnotes

^1^ https://www.psort.org/psortb/

^2^ https://dtu.biolib.com/DeepTMHMM
