# Supplementary material for: Looking for the mechanism of arsenate respiration of Fusibacter sp. strain 3D3, independent of ArrAB
Source: Front Microbiol. 2022 Dec 1;13:1029886. doi: 10.3389/fmicb.2022.1029886 (PMC9751042; doi:10.3389/fmicb.2022.1029886)
Supplement: Supplementary file 1 [file Data_Sheet_1.PDF]

SUPPLEMENTARY TABLES

Supplementary Table S1. Primers used to clone *arsC*-1 and *arsC*-2 gene from *Fas*.

Primers	Sequences 5'-3'	Annealing (°C)
arsC-1_*Hin*dIII_Rv	aagcttTCAATCTAAATTGTATTTGCTCTT	56.1
arsC-1_SHT_*Xho*I_Fw	ctcgagTatGAAGCCCGTTAAAATTTTA	
arsC-1_HT_*Xho*I_Fw	ctcgagATGAAGCCCGTTAAAATTTTA	
arsC-2_*Hin*dIII_Rv	aagcttTTATAAATCAAGCACAATTTG	60.0
arsC-2_SHT_*Xho*I_Fw	ctcgagtATGAGTAGAAAACCAAAAG	
arsC-2_HT_*Xho*I_Fw	ctcgagATGAGTAGAAAACCAAAAG	

Lowercase letters indicate changes made from the original sequence

Supplementary Table S2. Prediction of cell localization and presence of transmembrane regions in ArsC proteins.

	ArsC-1_Ecoli	ArsC_Staau	ArsC_Bacsu	ArsC_Rhime	ArsC-1_Fas	ArsC-2_Fas
**Localization***	Cytoplasmic	Cytoplasmic	Cytoplasmic	Cytoplasmic	Cytoplasmic	Cytoplasmic
**Transmembrane regions****	0	0	0	0	0	0

***** prediction made with PSORTb (Yu et al. 2010); ** prediction made with DeepTMHMM (Hallgren et al. 2022);
ArsC-1_Ecoli, *Escherichia coli* (Uniprot; P08692); ArsC_Staau, *Staphylococcus aureus* (Uniprot; P0A006), *Bacillus subtilis* (Uniprot; P45947), *Rhizobium meliloti* (Uniprot; Q92R44), *Fusibacter* sp. 3D3 (NCBI ArsC-1; WP_069871881, Arsc-2; WP_069871901)

Yu NY, Wagner JR, Laird MR, Melli G, Rey S, Lo R, Dao P, Sahinalp SC, Ester M, Foster LJ, Brinkman FS (2010) PSORTb 3.0: improved protein subcellular localization prediction with refined localization subcategories and predictive capabilities for all prokaryotes. *Bioinformatics* 26(13):1608-15. doi: 10.1093/bioinformatics/btq249.

Hallgren J, Tsirigos KD, Pedersen MD, Almagro Armenteros JJ, Marcatili P., Nielsen H., Krogh A, Winther O (2022) DeepTMHMM predicts alpha and beta transmembrane proteins using deep neural networks. *bioRxiv* 2022.04.08.487609. doi: 10.1101/2022.04.08.487609

Supplementary Table S3. BLAST results of predicted proteins of the Nqr complex.

Subsystem	Protein	Functional role	NCBI	Closest Protein Homology		
				**Species**	**UniProt**	**E-value**
**Oxidation-reduction process**	NqrB	Na(+)-translocating NADH-quinone reductase sub. B	GAU79379	*Finegoldia magna*	E1KXR0	5×10^{-115}
	NqrC	Na(+)-translocating NADH-quinone reductase sub. C	WP_069876132	*Clostridium ultunense*	M1ZGR7	9×10^{-56}
	NqrD	Na(+)-translocating NADH-quinone reductase sub. D	WP_069876131	*Finegoldia magna*	D6S727	5×10^{-91}
	NqrE	Na(+)-translocating NADH-quinone reductase sub. E	WP_175438433	*Psychromonas ingrahamii*	A1SSY7	6×10^{-65}
	NqrF	Na(+)-translocating NADH-quinone reductase sub. F	GAU79375	*Finegoldia magna*	B0S2C6	2×10^{-124}

Supplementary Table S4. Presence in *Fas* of genes expressing proteins related to ATP synthesis.

NCBI	Function (gene)	Closest reviewed (Swiss-Prot) protein			
		Identity	E-value	UniProt	Organism
WP_069870490	F0F1 ATP synthase subunit epsilon (*atpC*)	51.4%	$5.9x10^{-39}$	A8MJV8	*Alkaliphilus oremlandii* OhILAs
WP_069870492	F0F1 ATP synthase subunit beta (*atpD*)	76.9%	0.0	A8MJV9	*Alkaliphilus oremlandii* OhILAs
WP_069870494	ATP synthase F1 subunit gamma (*atpG*)	53.6%	$2.3x10^{-99}$	A6TK64	*Alkaliphilus metalliredigens* QYMF
WP_069870496	F0F1 ATP synthase subunit alpha (*atpA*)	75.5%	0.0	A8MJW1	*Alkaliphilus oremlandii* OhILAs
WP_069870498	F0F1 ATP synthase subunit delta (*atpH*)	37.8%	$3.8x10^{-35}$	A8MJW2	*Alkaliphilus oremlandii* OhILAs
WP_069870500	F0F1 ATP synthase subunit B (*atpF*)	48.4%	$1.3x10^{-46}$	Q0ZS23	*Clostridium paradoxum* JW-YL-7
WP_069870502	ATP synthase F0 subunit C (*atpE*)	86.4%	$2.7x10^{-45}$	A8MJW4	*Alkaliphilus oremlandii* OhILAs
WP_069870504	ATP synthase F0 subunit C (*atpE*)	71.4%	$2.6x10^{-34}$	A8MJW4	*Alkaliphilus oremlandii* OhILAs
WP_242877057	F0F1 ATP synthase subunit A (*atpB*)	only unreviewed entries available			
WP_069870509	ATP synthase subunit I (*atpI*)	only unreviewed entries available			

Accession numbers to NCBI and UniProtKB databases are under NCBI and UniProt columns, respectively.

Supplementary Table S5. Products of the genes located within the genetic contexts of Etfs in *Fas*. The *etf* gene context, EtfB group and NCBI accession numbers are indicated.

Microorganism	Context	Group	Protein accession numbers					
Fas	**1**	**G2B**	**FadR**<br>WP_069871746	**EtfB-1**<br>WP_069871747	**EtfA-1**<br>WP_069871749	**Ldh**<br>WP_069871751	**MalY**<br>WP_069871752	**Hyp1**<br>WP_069871754
	**2**	**G2A**	**Bcd**<br>WP_069875591	**EtfB-2**<br>WP_069875592	**EtfA-2**<br>WP_069875593			
***F. ferrireducens* Q10-2^{T}**	**1**	**G2B**	**FadR**<br>WP_194701605	**EtfB-1**<br>WP_194701606	**EtfA-1**<br>WP_194701607	**Ldh**<br>WP_194701608	**MalY**<br>WP_194701609	**Hyp1**<br>WP_194701610
	**2**	**G2A**	**Bcd**<br>WP_194702816	**EtfB-2**<br>WP_194702815	**EtfA-2**<br>WP_194702814			
***F. paucivorans* SEBR 4211^{T}**	**1**	**G2A**	**Bcd-1**<br>WP_213235038	**EtfB-1**<br>WP_213235037	**EtfA-1**<br>WP_213235036	**RocR**<br>WP_213235035		
	**2**	**G2A**	**Acd-2**<br>WP_213235641	**EtfB-2**<br>WP_213235640	**EtfA-2**<br>WP_213235639			
	**3**	**G2A**	**Acd-3**<br>WP_213236303	**EtfB-3**<br>WP_213236302	**EtfA-3**<br>WP_213236301	**Mfs**<br>WP_213236300		
	**4**	**G2C2**	**MhqN**<br>WP_213238184	**EtfB-4**<br>WP_213238183	**EtfA-4**<br>WP_213238182	**FixC**<br>WP_213238181	**FixX**<br>WP_213238180	
		**G2C2**		**EtfB-5**<br>WP_213238179	**EtfA-5**<br>WP_213238178			
	**5**	**G2C2**	**Acd**<br>WP_213238432	**EtfB-6**<br>WP_213238433	**EtfA-6**<br>WP_213238434			
	**6**	**G2C2**	**Acd**<br>WP_213238473	**EtfB-7**<br>WP_213238472	**EtfA-7**<br>WP_213238471	**HisJ**<br>WP_213238470		
***Fusibacter* sp. A1**	**1**	**G2A**	**Acd**<br>WP_129487661	**EtfB**<br>WP_129487660	**EtfA**<br>WP_129487659	**MaoC**<br>WP_129487658	**YciA**<br>WP_129487657	
***F. tunisiensis* BELH1^{T}**	**1**	**G2A**	**Acd**<br>WP_204662590	**EtfB**<br>WP_204662592	**EtfA**<br>WP_204662595	**Hyp**<br>WP_204662597		

SUPPLEMENTARY FIGURES

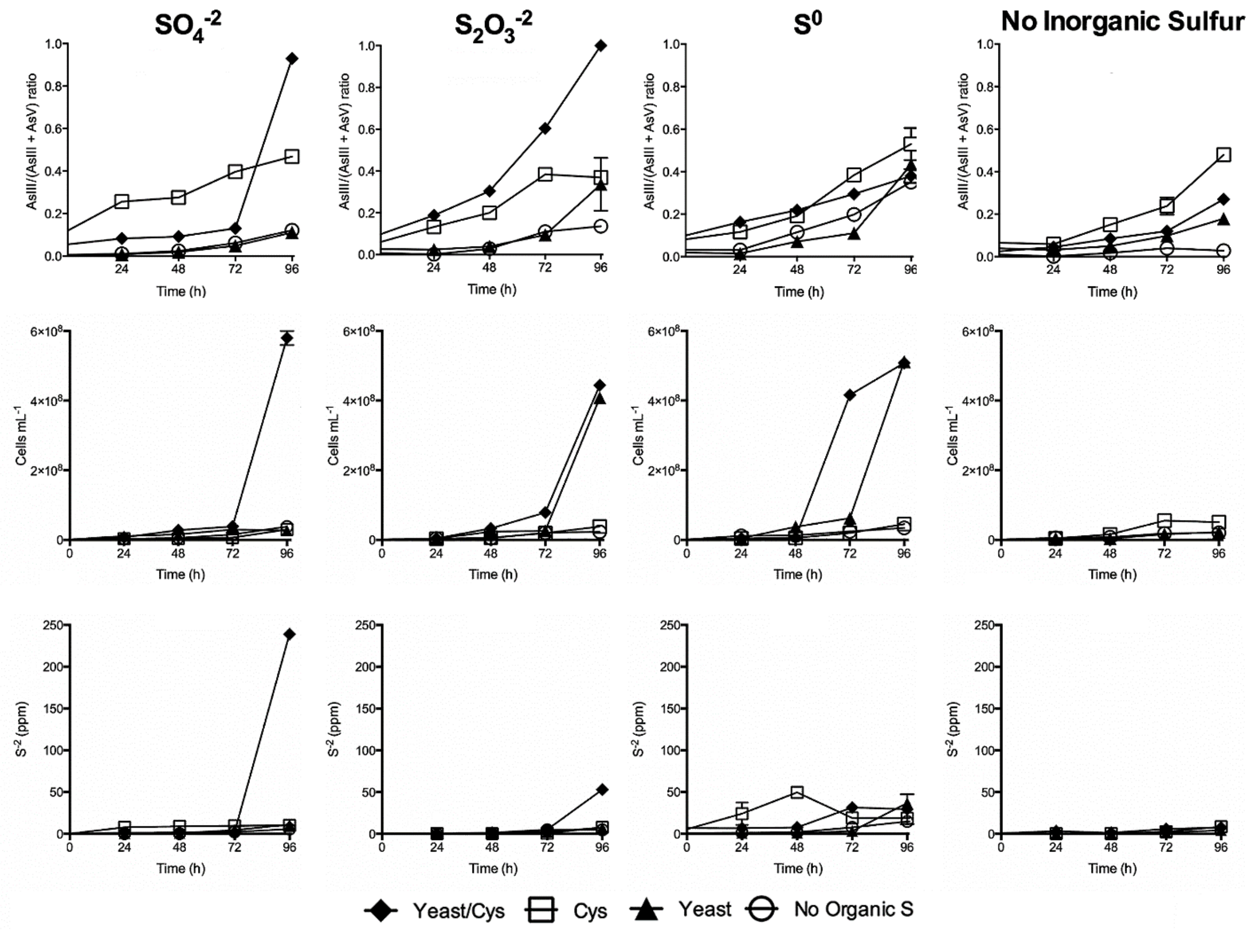

Supplementary Figure S1. *Fas* cultures amended with different inorganic and organic sulfur sources. Sodium sulfate (SO_4^{-2}), sodium thiosulfate ($S_2O_3^{-2}$), mineral sulfur (S°) and no inorganic sulfur sources were tested, with yeast extract/cysteine (filled rhombus), cysteine (empty square), yeast extract (filled triangle), and no organic sulfur sources (empty circle). Graphs show arsenic ratio (AsIII/[AsIII + AsV]), cell number (cells mL^{-1}) and S^{-2} production (ppm) in cultures performed in AsV (2 mM) Newman's media with $1x10^6$ initial cells and incubated at 30 ºC in an anaerobic chamber under non-stirring conditions. Error bars represent the standard error of triplicate cultures.

```
WP_069871901-ArsC2      MSRKPKVAFI--CVHNSCRSQMAEALGKHFAGDVFESY-SAGTEMKPQINQDAVRLIKDL  }
P0A006                  -MDKKTIYFI--CTGNSCRSQMAEGWGKEILGEGWNVY-SAGIETHG-VNPKAIEAMKEV  } ArsC Trx
WP_069871881-ArsC1      -MKPVKILFV--CVHNSARSQMAEAFLNDY-GAAFAIAESAGIE-KGTLNPLAVKVMDEI  }
PAX80313                ---MITLYGIKNCDTIKKARRWLEDNGVDYRFHDYRVDGLDNALLHAFISELGW---EAL
PAX80297                MSDAIKIYHNPRCSKSRETLELLKSNGVDPEVVLYLETPADATTLRELLQMLGMSSAREL  }
P0AB96                  -MSNITIYHNPACGTSRNTLEMIRNSGTEPTIIHYLETPPTRDELVKLIADMGI-SVRAL  } ArsC Glx
PAX80519                -MSNITIYHNPACGTSRNTLEMIRNSGNEPTIIYYLDTPPTRDELTKLISDMGI-SVRAL  }
                             .:     *       .  .    .     :             :   .      :

WP_069871901-ArsC2      YKIDMEKTQSSKLLTEIP------EVDIVIKMGCNVICPFLPSQYEAD-WGLDD------
P0A006                  -DIDISNHTSDLIDNDILKQSD--LVVTLCSDAD-NNCPILPPNVKKEHWGFDD------
WP_069871881-ArsC1      -GIDISKNEVNSVFEFFKNHKLYTYVVTVCDESSGQKCPIFPGVREMIHWSLDD------
PAX80313                LNTRGTTWRKLDEATRNQITDASSAAALMIEMPAIIKRPLLCAPGKPMLLGFSE------
PAX80297                MRQKEDLYKSLNLADVNLSEDA--LIQAMVENPKLMERPIVVAKGQARIGRPPE------
P0AB96                  LRKNVEPYEELGLAEDKFTDDR--LIDFMLQHPILINRPIVVTPLGTRLCRPSEVVLEIL
PAX80519                LRKNVEPYEQLGLDEDKFSDEQ--LIDFMIQHPILINRPIVVTPLGTRLCRPSEIVLDIL
                                                    : .       *:.            :

WP_069871901-ArsC2      PSGKSDEAFKL------IIDKIEANVKLLADQIRTQQIVLD
P0A006                  PAGKEWSEFQR------VRDEIKLAIE---------KFKLR
WP_069871881-ArsC1      PSSFEGTEEERLEKTRVVRDQIKANVLGLIEDLK-SKYNLD
PAX80313                ------SSYTQ------FFNEV-------------------
PAX80297                ----------Q------VLEIIG------------------
P0AB96                  PDAQKGAFSKEDGEK--VVDEAGKRLK--------------
PAX80519                PEGQKGSFTKEDGEK--VIDETGKRVK--------------
                                         . :
```

Supplementary Figure S2. Multiple alignment of aminoacid sequences of known and putative arsenate reductases dependent of glutaredoxins (Glx) or thioredoxin (Trx). *E. coli* strain K12 (P0AB96), *Staphylococcus aureus* (P0A006), *Fas* (WP_069871881, ArsC-1 and WP_069871901, ArsC-2) and *Citrobacter* sp. TSA-1 (PAX80297; PAX80313 and PAX80519).

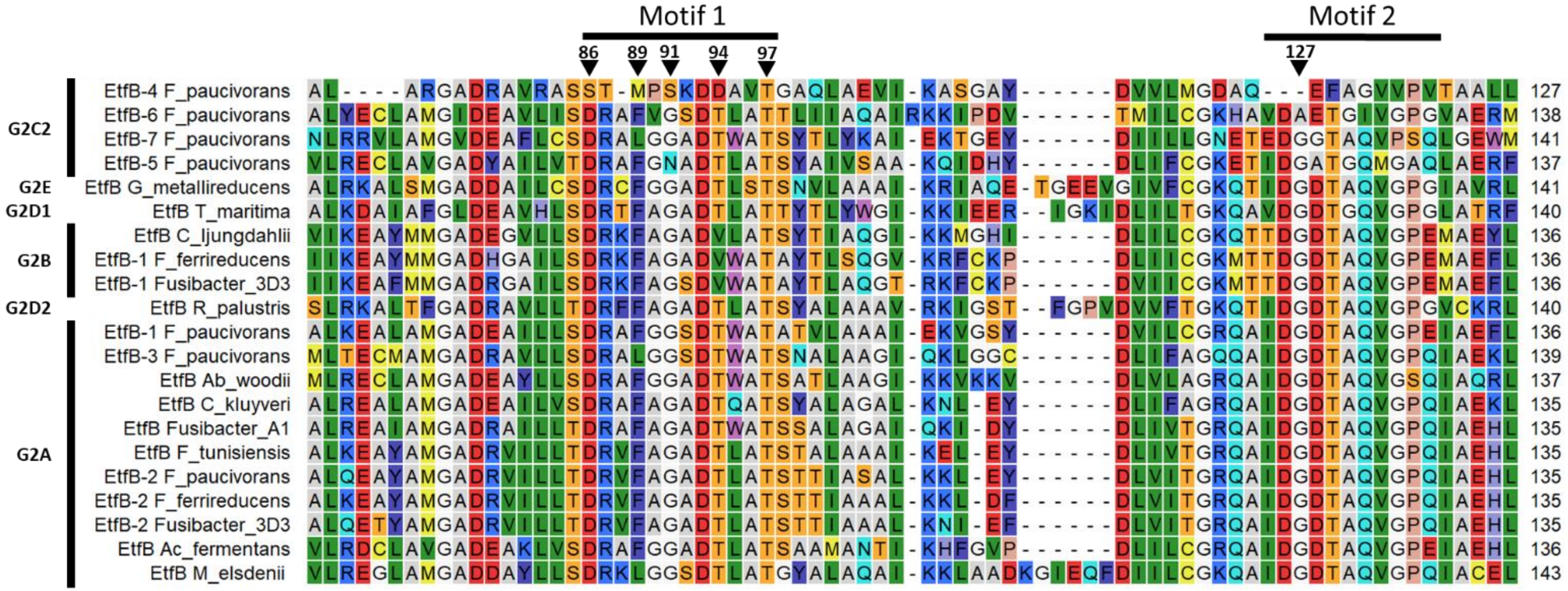

Supplementary Figure S3. Protein sequence alignment to compare characterized electron-transferring flavoproteins with those from *Fusibacter*. The horizontal bars indicate motifs 1 and 2, corresponding to the NADH- and FAD-binding sites in bifurcating Etfs, respectively. The inverted triangles indicate residues proposed to coordinate NADH and FAD and the numeration corresponds to EtfB *R_palustris*. The Etf groups are indicated on the left side. Representatives of the EtfB groups are from *Geobacter metallireducens* GS-15 (*G_metallireducens*; YP_383650), *Thermotoga maritima* MSB8 (*T_maritima*; NP_229330), *Clostridium ljungdahlii* PETCPETC (*C_ljungdahlii*; YP_003780321), *Rhodopseudomonas palustris* BisA53 (*R_palustris*; YP_783418), *Acetobacterium woodii* WB1 (*Ab_woodii*; AFA48355), *Clostridium kluyveri* DSM 555 (*C_kluyveri*; YP_001393858), *Acidaminococcus fermentans* VR4 (*Ac_fermentans*; YP_003398269), *Megasphaera elsdenii* T81 (*M_elsdenii*; WP_022498188). *Fusibacter* proteins are listed in Table S5. The residues were colored according to the RasMol amino color scheme that colors amino acids according to traditional amino acid properties.

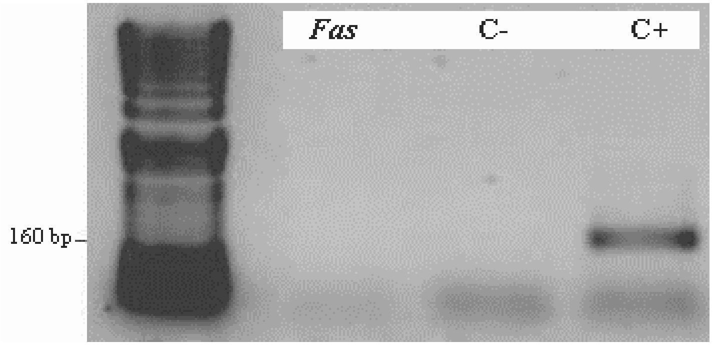

Supplementary Figure S4. Confirmation of the absence of the *arrAB* gene cluster in *Fas*. PCR amplification using the primers arrAf and arrAr to target a ~160–200 bp fragment of *arrA* gene was performed (Malasarn et al., 2004). The negative control (C-) does not contain DNA template and, as positive control (C+), *Shewanella* sp. strain ANA-3 DNA was used.

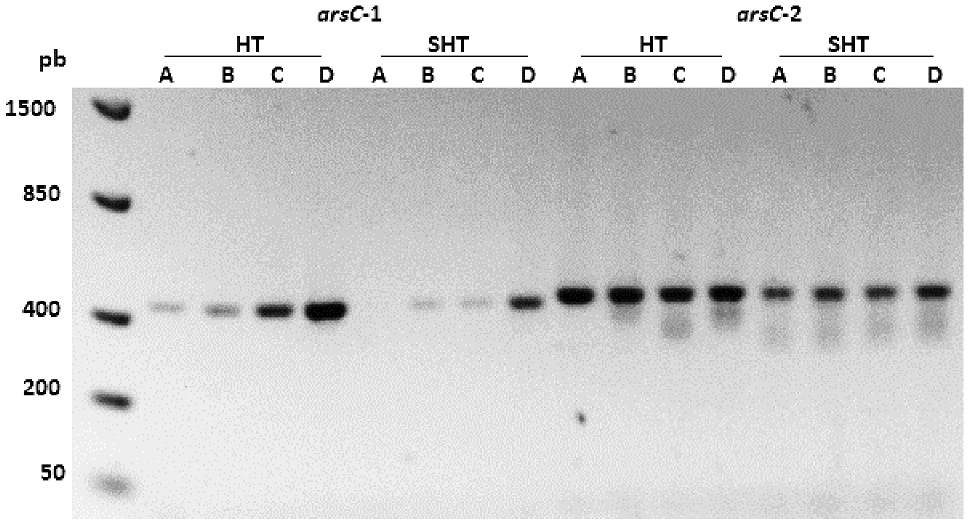

Supplementary Figure S5. PCR amplification of the arsenate reductase genes *arsC*-1 and *arsC*-2 from *Fas* genomic DNA at different hybridization temperatures. (A) 60.0, (B) 59.2, (C) 58.0, and (D) 56.1 °C. The DNA sizes (bp) are indicated on the left side of the DNA ladder (first lane). SHT refers to products without His-tag, and HT to those with His-tag.

Malasarn D, Saltikov CW, Campbell KM, Santini JM, Hering JG, Newman DK. (2004) *arrA* is a reliable marker for As(V) respiration. *Science* 306(5695), 455. doi: 10.1126/science.1102374.

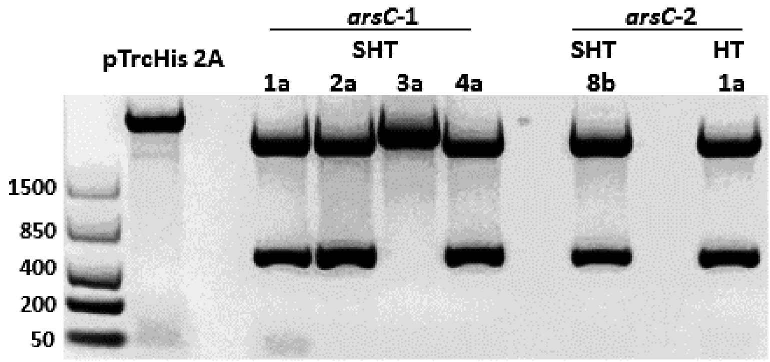

Supplementary Figure S6. Releasing of *arsC*-1 and *arsC*-2 DNA fragments from pGEM-T vector. DNA fragments were released from different recombinant clones by digestion of DNA with *Xho*I and *Hind*III and purified from the agarose gel. The plasmid pTrcHis 2A was digested by the same restriction enzymes to allow the directional insertion of the *arsC* fragments. The DNA sizes (bp) are indicated in the left side of the DNA ladder (first lane).

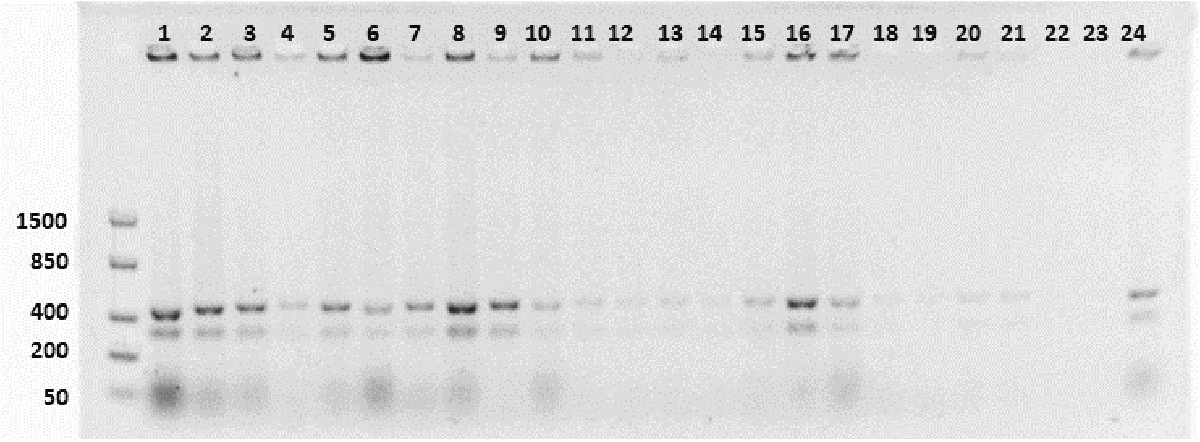

Supplementary Figure S7. Determination of the presence of the insert arsC-1Fas SHT on the plasmid pTrcHis2A by colony PCR. The number on the head of each lane identifies the *E. coli* WC3110 recombinant clone. Although not clearly seen in the photograph, all the 24 tested clones were positive. The DNA sizes (bp) are indicated in the left side of the DNA ladder lane.

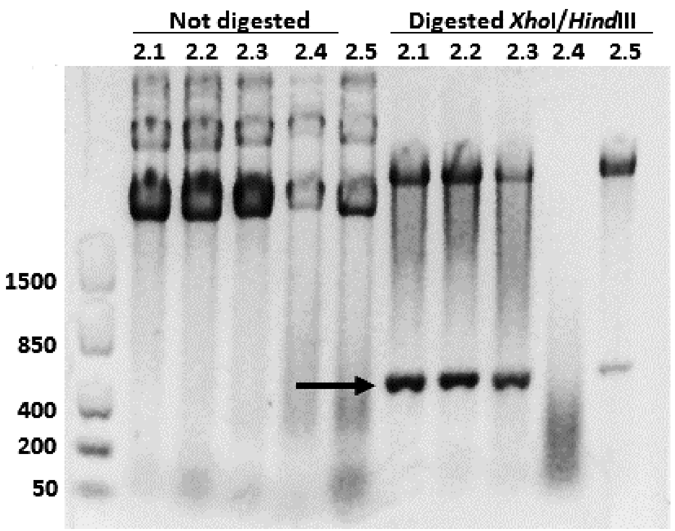

Supplementary Figure S8. Determination of the presence of the insert *arsC*-2$_{Fas}$ SHT by digestion of the plasmid pTrcHis2A. The number on the head of each lane identifies the *E. coli* WC3110 recombinant clone from which the plasmid was obtained. The DNA sizes (bp) are indicated in the left side of the DNA ladder lane.
